# Spatial distribution of G6PD deficiency variants across malaria-endemic regions

**DOI:** 10.1186/1475-2875-12-418

**Published:** 2013-11-15

**Authors:** Rosalind E Howes, Mewahyu Dewi, Frédéric B Piel, Wuelton M Monteiro, Katherine E Battle, Jane P Messina, Anavaj Sakuntabhai, Ari W Satyagraha, Thomas N Williams, J Kevin Baird, Simon I Hay

**Affiliations:** 1 Spatial Ecology and Epidemiology Group, Department of Zoology, University of Oxford, South Parks Road, Oxford, UK; 2 Eijkman-Oxford Clinical Research Unit, Jalan Diponegoro No. 69, Jakarta, Indonesia; 3 Evolutionary Ecology of Infectious Disease Group, Department of Zoology, University of Oxford, South Parks Road, Oxford, UK; 4 Tropical Medicine Foundation Dr. Heitor Vieira Dourado, Manaus, Amazonas, Brazil; 5 University of the State of Amazonas, Manaus, Amazonas, Brazil; 6 Institut Pasteur, Unité de Génétique Fonctionnelle des Maladies Infectieuses, Paris, France; 7 Eijkman Institute for Molecular Biology, Jakarta, Indonesia; 8 Kenya Medical Research Institute/Wellcome Trust Programme, Centre for Geographic Medicine Research-Coast, Kilifi District Hospital, Kilifi, Kenya; 9 Department of Medicine, Imperial College, St Mary’s Hospital, London, UK; 10 Centre for Tropical Medicine, Nuffield Department of Clinical Medicine, University of Oxford, Oxford, UK

**Keywords:** Glucose-6-phosphate dehydrogenase deficiency, G6PD, Genetic variants, Spatial distribution, Primaquine, *Plasmodium vivax*, Malaria elimination, Haemolysis

## Abstract

**Background:**

Primaquine is essential for malaria control and elimination since it is the only available drug preventing multiple clinical attacks by relapses of *Plasmodium vivax.* It is also the only therapy against the sexual stages of *Plasmodium falciparum* infectious to mosquitoes, and is thus useful in preventing malaria transmission. However, the difficulties of diagnosing glucose-6-phosphate dehydrogenase deficiency (G6PDd) greatly hinder primaquine’s widespread use, as this common genetic disorder makes patients susceptible to potentially severe and fatal primaquine-induced haemolysis. The risk of such an outcome varies widely among *G6PD* gene variants.

**Methods:**

A literature review was conducted to identify surveys of *G6PD* variant frequencies among representative population groups. Informative surveys were assembled into two map series: (1) those showing the relative proportions of the different variants among G6PDd individuals; and (2) those showing allele frequencies of *G6PD* variants based on population surveys without prior G6PDd screening.

**Results:**

Variants showed conspicuous geographic patterns. A limited repertoire of variants was tested for across sub-Saharan Africa, which nevertheless indicated low genetic heterogeneity predominated by the *G6PD A-*^*202A*^ mutation, though other mutations were common in western Africa. The severe *G6PD Mediterranean* variant was widespread across western Asia. Further east, a sharp shift in variants was identified, with high variant heterogeneity in the populations of China and the Asia-Pacific where no single variant dominated.

**Conclusions:**

*G6PD* variants exhibited distinctive region-specific distributions with important primaquine policy implications. Relative homogeneity in the Americas, Africa, and western Asia contrasted sharply with the heterogeneity of variants in China, Southeast Asia and Oceania. These findings will inform rational risk assessments for primaquine in developing public health strategies for malaria control and elimination, and support the future development of regionally targeted policies. The major knowledge gaps highlighted here strongly advocate for further investigation of *G6PD* variant diversity and their primaquine-sensitivity phenotypes.

## Background

The discovery of glucose-6-phosphate dehydrogenase deficiency (G6PDd) occurred during the clinical development of primaquine against the relapse of *Plasmodium vivax* malaria in American prisoner volunteers during and immediately after the Second World War [1]. The high prevalence of this inherited disorder among people at risk of malaria [2], along with the practical difficulty of its diagnosis where almost all of them live [3], sharply limits the otherwise enormous public health importance and utility of primaquine in controlling and eliminating malaria [4-8]. This drug is currently the only licensed therapy active against the latent liver-stages of *P. vivax* responsible for multiple relapses in the weeks, months, and several years following a single infectious bite by an anopheline mosquito [9]. As with the primary infection, and despite the long-held dogma that considered vivax malaria clinically benign, each relapse carries risk of severe disease and mortality [5]. Furthermore, primaquine is the only drug with activity against the mature transmission-stages of all *Plasmodium* species [4,10,11], giving it a role of undeniable importance in reducing transmission levels, most particularly in preventing the spread of artemisinin-resistant *Plasmodium falciparum*[12-15]. The recently published model-based geostatistical mapping study showed that G6PDd is prevalent across malaria-endemic countries, with an estimated median allele frequency in these regions of 8.0% (50% CI: 7.4-8.8) [2], thought to be driven by a selective advantage against life-threatening malaria [16-18]. Despite its prevalence and hindrance to malaria control, the spatial patterns of the genetically and clinically diverse *G6PD* mutations are poorly documented globally; this knowledge gap, highlighted by others [19], constitutes the focus of the present study. A better understanding of the spatial epidemiology of *G6PD* gene variants would support assessments towards safe delivery strategies to increase access to primaquine.

G6PD enzyme generates nicotinamide adenine dinucleotide phosphate (NADPH), which represents the primary defence against oxidative stresses in red blood cells (RBC). Mutations in the *G6PD* gene can destabilize the enzyme and reduce its activity levels, leaving cells vulnerable to damage from exogenous triggers, including certain foods, infections, and a range of drugs, that may lead to RBC lysis and acute haemolytic anaemia (AHA) [20,21]. The clinical burden of G6PDd was evaluated in the Global Burden of Disease Study 2010 [22,23], and includes a range of pathologies, notably neonatal jaundice and AHA. The clinical symptoms due to primaquine-induced haemolysis range from negligible to lethal [24,25] depending largely upon the variants involved and drug dosing.

At least 186 mutations have been characterized in the *G6PD* gene [26], although not all are polymorphic and of clinical significance. *G6PD* variants have been categorized into three types (Table 1) [21,25]. About half of the known variants appear to be sporadic mutations identified in only a handful of patients [27]. These rare variants, classified as Type 1, usually express very severe chronic disease – a pathology known as chronic non-spherocytic haemolytic anaemia (CNSHA) – which can result in lifelong dependency on blood transfusion [25]. Although numerous by type, these variants never reach polymorphic frequencies (prevalence ≥1%) and are thus not of serious public health concern. Type 2 variants, in contrast, do reach frequencies ≥1% and put individuals at significant risk of haemolytic crises. These Type 2 variants correspond to the Class II and III variants of the WHO-endorsed classification system [28,29]. Given their public health significance, being both polymorphic and clinically significant, Type 2 variants are the focus of the present study. Type 3 variants express phenotypically normal G6PD activity and present no clinical or public health concerns.

**Table 1:** G6PDd variant types

**Type**	**Residual enzyme activity**	**Population-level prevalence**	**Clinical significance**	**Primaquine-associated public health concern**
Type 1	<10%	Sporadic (never polymorphic)	Severe and chronic: CNSHA	None – too rare
Type 2^§^	<1-50%	Polymorphic	Asymptomatic until exposed to an exogenous trigger	Yes – commonly inherited and cause individuals to be at risk of haemolysis
Type 3	Normal (>50%)	Polymorphic (wild-type)	None	None – G6PD normal

^§^Type 2 variants have previously been separated into Class II and Class III variants of the WHO-endorsed G6PDd variant classification [28,29]. For many variants, however, this subdivision is blurred and poorly defined [25].

The *G6PD A-* variant from Africa has been the most thoroughly studied with respect to sensitivity to primaquine. This may be a consequence of being the first studied and the most accessible (in African American volunteers) during primaquine development [30]. This variant typically exhibits residual enzyme activity of about 5-10% of normal levels [31]. Primaquine toxicity in the *G6PD A-* volunteers was relatively mild and self-limiting [32]; *G6PD A-* individuals dosed with daily primaquine for four months have been observed to typically recover from relatively shallow AHA within about three weeks, despite continued daily dosing [33]. In contrast, the *G6PD Mediterranean* variant, which is common across southern Italy (particularly Sardinia) and the Arabian Peninsula [34], exhibits exceedingly low residual enzyme activity (<1% [35]) and predisposes individuals to favism [36,37] and clinically severe AHA following primaquine therapy [12,35,38,39]. *G6PD Mahidol* is the best-characterized Asian variant and is considered predominant across Myanmar and Thailand [40,41]. Enzyme activity in *G6PD Mahidol* individuals is reduced to 5-32% of normal levels [42], and its primaquine sensitivity phenotype lies between that of the *G6PD A-* and *Mediterranean* variants [43,44]. As for the balance of many dozens of other *G6PD* variants, residual enzyme activity is generally known for many [26], but the primaquine sensitivity phenotypes for any of these remains effectively unknown [4]. There may be an inverse correlation between residual enzyme activity and AHA sensitivity to primaquine (as roughly seen with the three variants discussed), but this is not yet firmly established.

The diversity of G6PDd phenotypes and genotypes, and our limited knowledge thereof, greatly compounds the difficulty of addressing the technical and practical limitations which this deficiency imposes on primaquine treatment for attacking the endemic malarias [45]. National authorities responsible for the prevention, control and treatment of endemic malaria naturally strive for evidence-based practices that minimize risk and maximize benefit. The complexity of the problem imposed by G6PDd – both by its genetic variability and its heterogeneous prevalence [2] – compounds the difficulty of developing practical and useful tools for assessing risk and benefit. The present study begins the task of characterizing *G6PD* variant spatial distribution and diversity, with the aim of contributing towards the evidence-based policies for primaquine treatment so essential to realizing the vision of malaria elimination.

## Methods

The aim of this study was to map population surveys reporting the prevalence of the *G6PD* variants of greatest public health significance. A systematic literature review was conducted of online biomedical databases for all records referring to “G6PD” or “glucose-6-phosphate dehydrogenase” and then cross-referenced with previously published *G6PD* variant databases [26,46-50]. The study was limited to data from malaria-endemic countries [2,51,52], corresponding to where primaquine therapy is needed. Refer to Additional file 1 for more detailed descriptions of the methodology. The assembled datasets are available from the authors on request.

### Survey inclusion criteria

**Figure 1:**
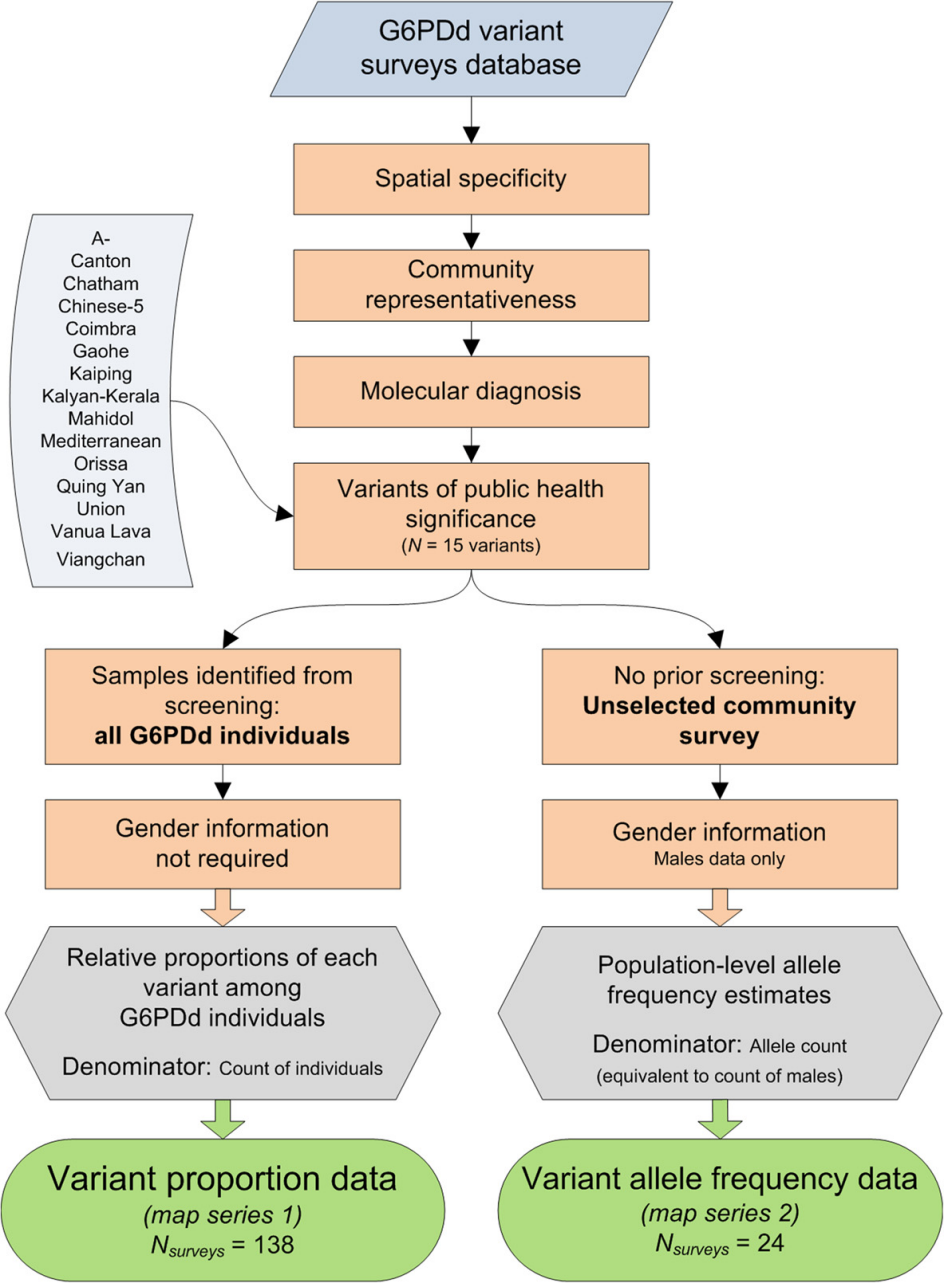
**Survey inclusion criteria and G6PDd variant map outputs.** Orange rectangles indicate the exclusion criteria, grey hexagons summarize the two final input data, and green rods represent the two map types.

The assembled library of sources was reviewed for population surveys of *G6PD* variants and those meeting a specific set of inclusion criteria (Figure 1) were identified. First, only surveys that could be geopositioned to at least the national level were included, and these were mapped to the highest spatial resolution available, ideally as point locations (e g, villages). Second, to ensure that survey samples were widely representative of the communities being studied, only surveys which provided apparently unbiased prevalence estimates were included: all case studies and patient groups, malaria patients (to avoid underestimating frequencies of *G6PD* variants which may provide a protective effect against malaria), family studies, and samples selected according to ethnic background were therefore excluded. Third, only surveys using molecular diagnostics were included to avoid the diagnostic uncertainty of surveys reliant on biochemical methods (see Additional file 1).

Surveys meeting these criteria were of two types depending upon the nature of the population samples surveyed and were mapped as two separate series:

### Map series 1: variant proportion maps

Studies examining individuals who had been previously identified as phenotypically G6PDd from a population screening survey and were being followed-up for molecular diagnosis of underlying mutations were included in map series 1. Pie charts were chosen to display the relative proportions of the different variants identified in each of the surveys, with each colour-coded segment proportional to the variant’s relative frequency across the study sample. Sample size was reflected in the relative sizes of the pie charts, which was transformed on a square-root scale to allow clear visualization.

It is important to note that studies did not always attempt to identify all the variants represented in the legends of the maps. For instance, if only *G6PD Mediterranean* was tested for among a sample of deficient individuals, those individuals who tested negative would be included in the “Other” category, regardless of whether they expressed one of the other variants listed in the legend or a completely different one. The absence of a specific variant from a sample can only be inferred if no “Other” samples are reported.

### Map series 2: allele frequency maps

Surveys which investigated *G6PD* variants in cross-sectional population samples without prior phenotype screening were included in map series 2. These studies estimated the allele frequencies of selected *G6PD* variants at the population level and were mapped spatially using bar charts to represent the allele frequencies. This visualization conveyed the important concept that frequency estimates are only available for variants that were included in the diagnoses, underlining the importance of knowing which variants had been tested for. The variants tested for were named in the vertical bars of the bar charts. Empty spaces along the *x* -axis indicate that the named variant was tested for but not identified in the population sample. The size of the plots meant that in some cases, significant spatial uncertainty was introduced to their positioning on the map (Additional file 1).

The *G6PD* gene’s X-linked inheritance means that allele frequencies correspond to frequencies among males. Heterozygous inheritance in females makes allele frequency estimates from female samples harder to calculate confidently as not all studies consistently distinguished heterozygotes from homozygotes. For reliability, therefore, only data from males were included in these variant allele frequency maps.

### Variant inclusion criteria

Given the diversity of *G6PD* variants, for the purposes of this mapping study it was necessary to identify those variants that presented a primary public health concern. This study focussed on Type 2 variants which (i) express significantly reduced enzyme activity (<50%); (ii) are clinically significant by predisposing individuals to haemolytic anaemia; and, (iii) reach polymorphic allele frequencies at the population level (Table 1). Although 77 Type 2 variants have so far been described [26], the database of population survey data compiled in this study indicated that most variants were reported fewer than ten times (most fewer than three) and, when identified, were found to have low prevalence. A clear subset of variants was responsible for the majority of investigated G6PDd cases (Additional file 1); these were defined for the purposes of this study to be those variants reported from a minimum of ten localities across the malaria-endemic region. This ensured that the spatial distributions of the most significant variants could be legibly visualized in the maps. Fifteen variants met these criteria: *G6PD A-, Canton, Chatham, Chinese-5, Coimbra, Gaohe, Kaiping, Kalyan-Kerala, Mahidol, Mediterranean, Orissa, Quing Yan, Union, Vanua Lava*, and *Viangchan* (Figure 1).

## Results

### The database

A total of 18,939 bibliographic sources were identified from keyword searches, of which 2,176 were considered likely to include spatial information about *G6PD* variants. Following their detailed review, 93 published and unpublished sources were identified which reported surveys eligible for inclusion in the maps (listed in the Additional file 2). From these sources (which could each report several surveys from multiple locations), 138 population surveys could be geolocated and met the criteria for map series 1, as these were of community samples which had undergone prior screening for phenotypic G6PDd. Map series 2 was populated with 24 geolocated population surveys of community samples providing variant frequency estimates. Map series 1 data were predominantly from populations in Asia (123/138 surveys; 89%), while map series 2 data were almost exclusively from the Africa + region (Africa, Yemen and Saudi Arabia [53]: 20/24 surveys, or 83%). These database descriptors are summarized in Table 2.

**Table 2:** Summary of input data according to map type

		**Africa+**	**Americas**	**Asia**	**Global**
		***(Malaria-endemic countries only)***			
***Map series 1*** Variant proportion maps	*N*_surveys_	6	9	123	138
	*N*_countries_	5	4	16	25
	*N*_G6PDd indivs_	258	636	5,278	6,172
	Mean sample size	43	71	43	45
***Map series 2*** Variant frequency maps	*N*_surveys_	20	1	3	24
	*N*_countries_	13	1	2	16
	*N*_male__indivs_	6,796	509	448	7,753
	Mean sample size	340	509	149	323

### Distribution of *G6PD* variants in malaria-endemic regions

**Figure 2:**
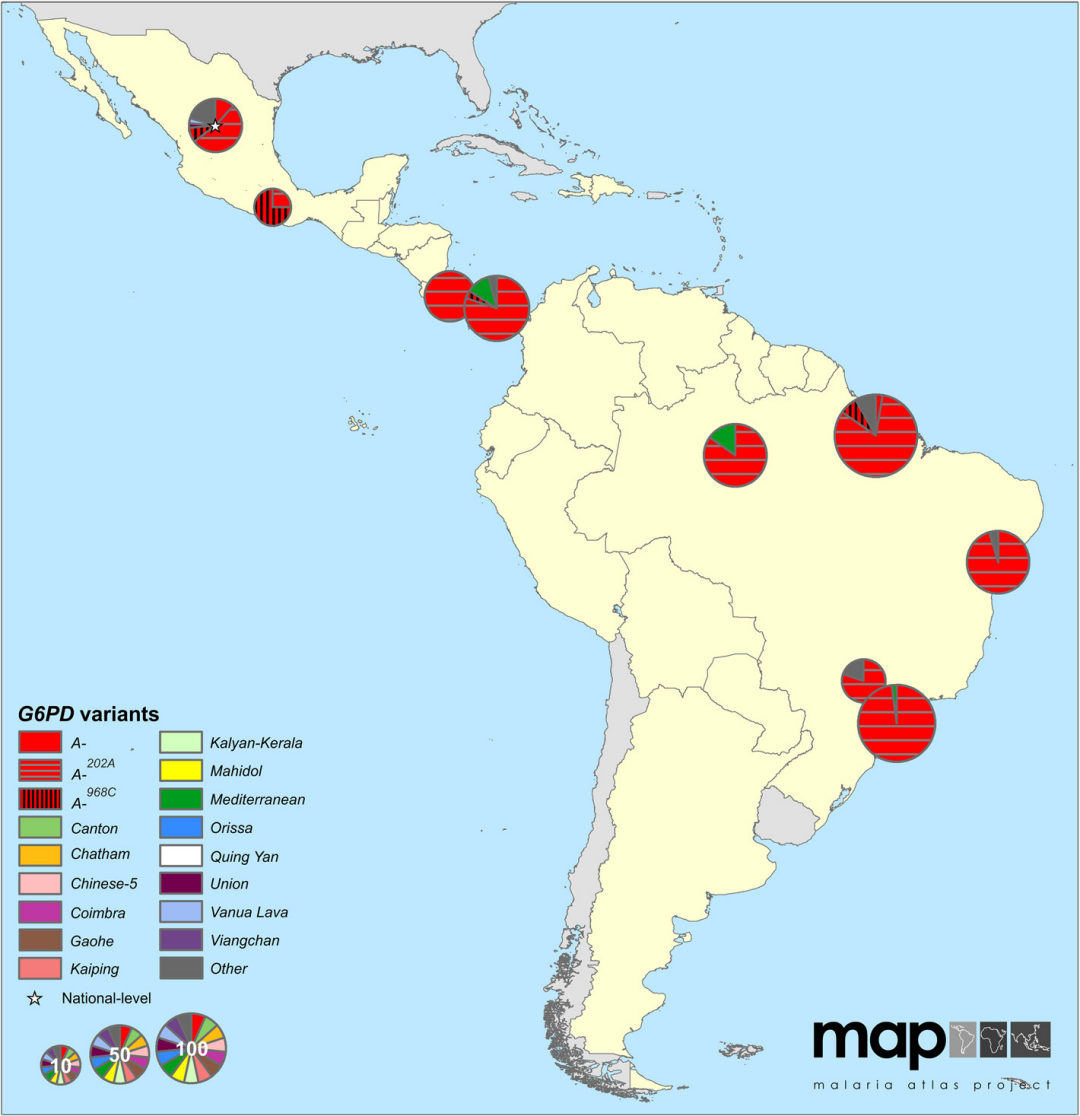
**G6PDd variant proportion map: Americas (map series 1).** Map includes nine surveys with a mean sample size of 71 (range: 8-196); one survey was mapped at the national level. Pie charts represent individuals previously identified as G6PDd. Sample size is reflected in the size of the pie charts, which is transformed for clarity on a square-root scale. Surveys which could only be mapped to the country-level are indicated by a white star; this applied to seven surveys globally. Spatial duplicates from independent studies were mapped with a “jitter” of 0.5-1 decimal degrees in their latitude or longitude coordinates to allow visualization of the multiple charts. Malaria-endemic countries in the region mapped are shown with a yellow background; white backgrounds indicate endemic countries outside the region in focus; grey backgrounds represent malaria-free countries. G6PDd variants that could not be diagnosed were reported as “Other”.

**Figure 3:**
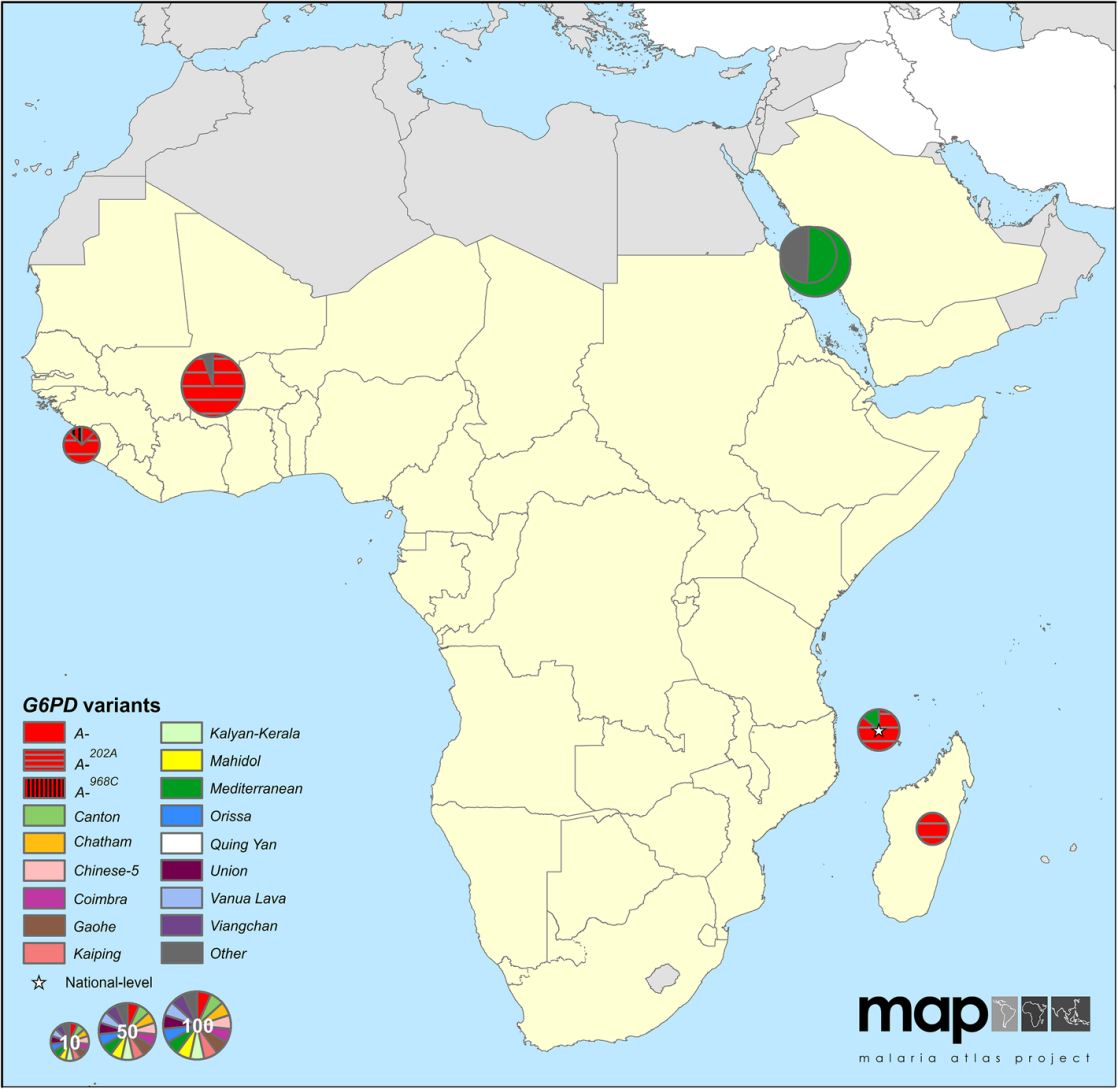
**G6PDd variant proportion map: Africa+ (map series 1).** Map includes six surveys with a mean sample size of 43 (range: 5-110). One survey was mapped at the national level. (See Figure 2 for full legend).

**Figure 4:**
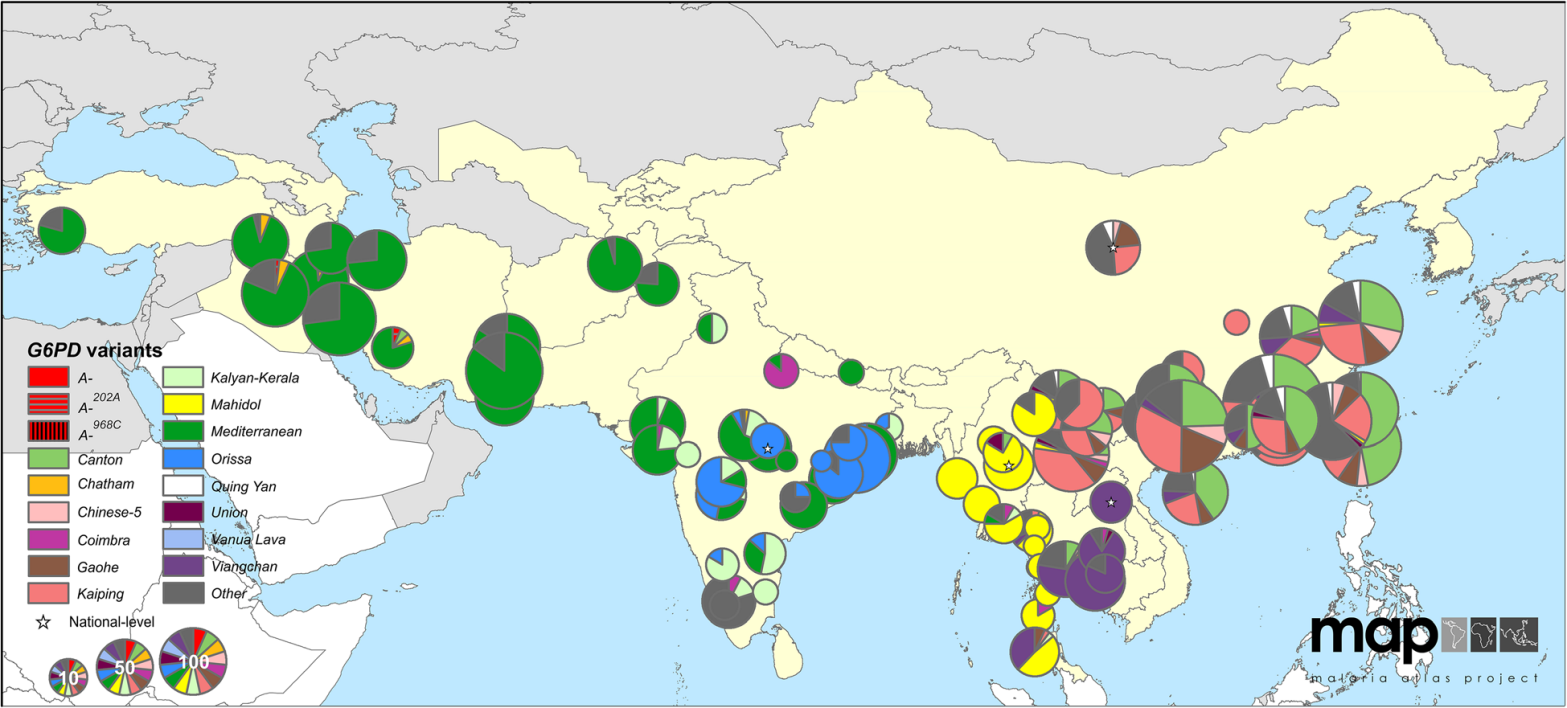
**G6PDd variant proportion map: Asia (map series 1).** Map includes 87 surveys with a mean sample size of 53 (range: 1-532). Four surveys were mapped to the national level. (See Figure 2 for full legend).

**Figure 5:**
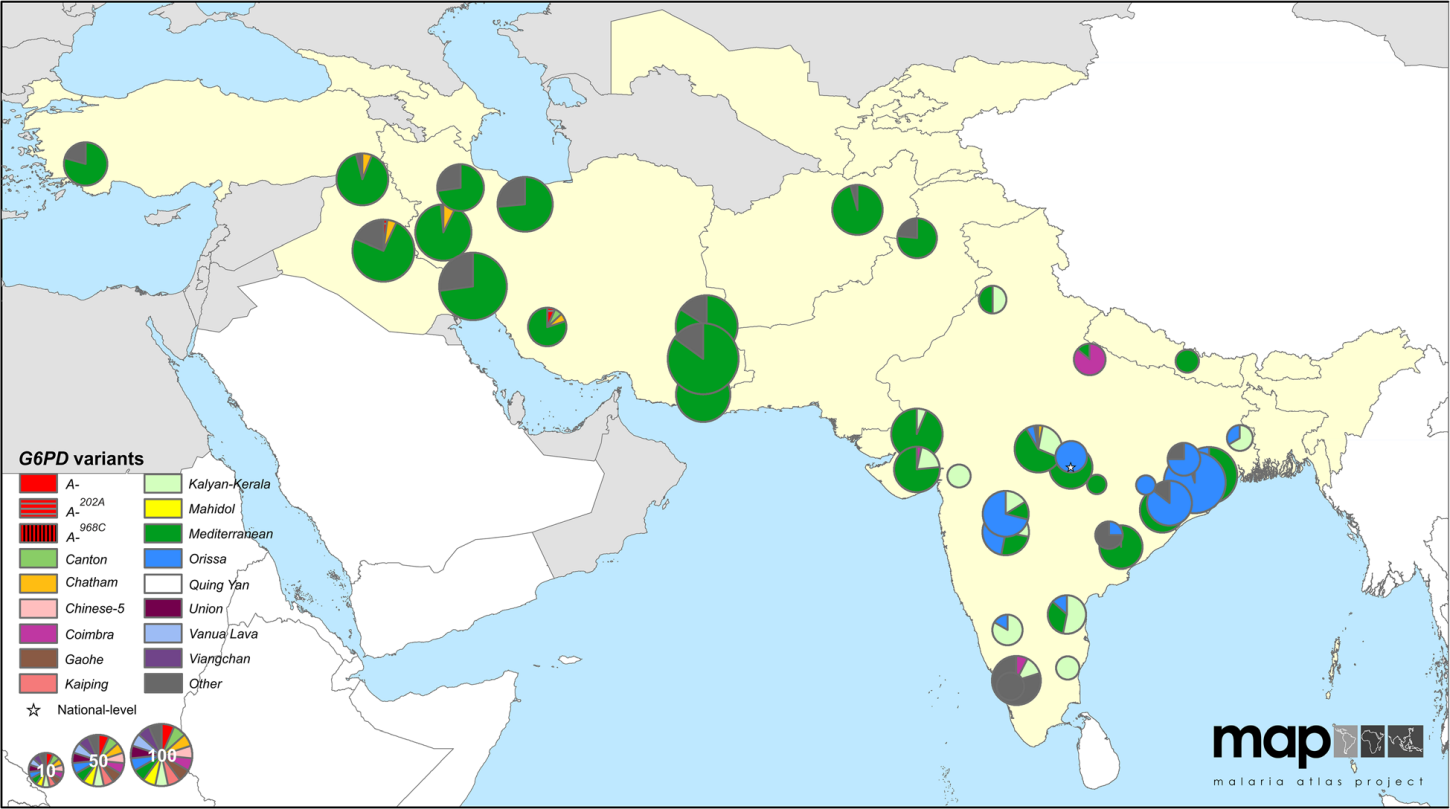
**G6PDd variant proportion map: West Asia (map series 1).** A higher resolution map of Figure 4.

**Figure 6:**
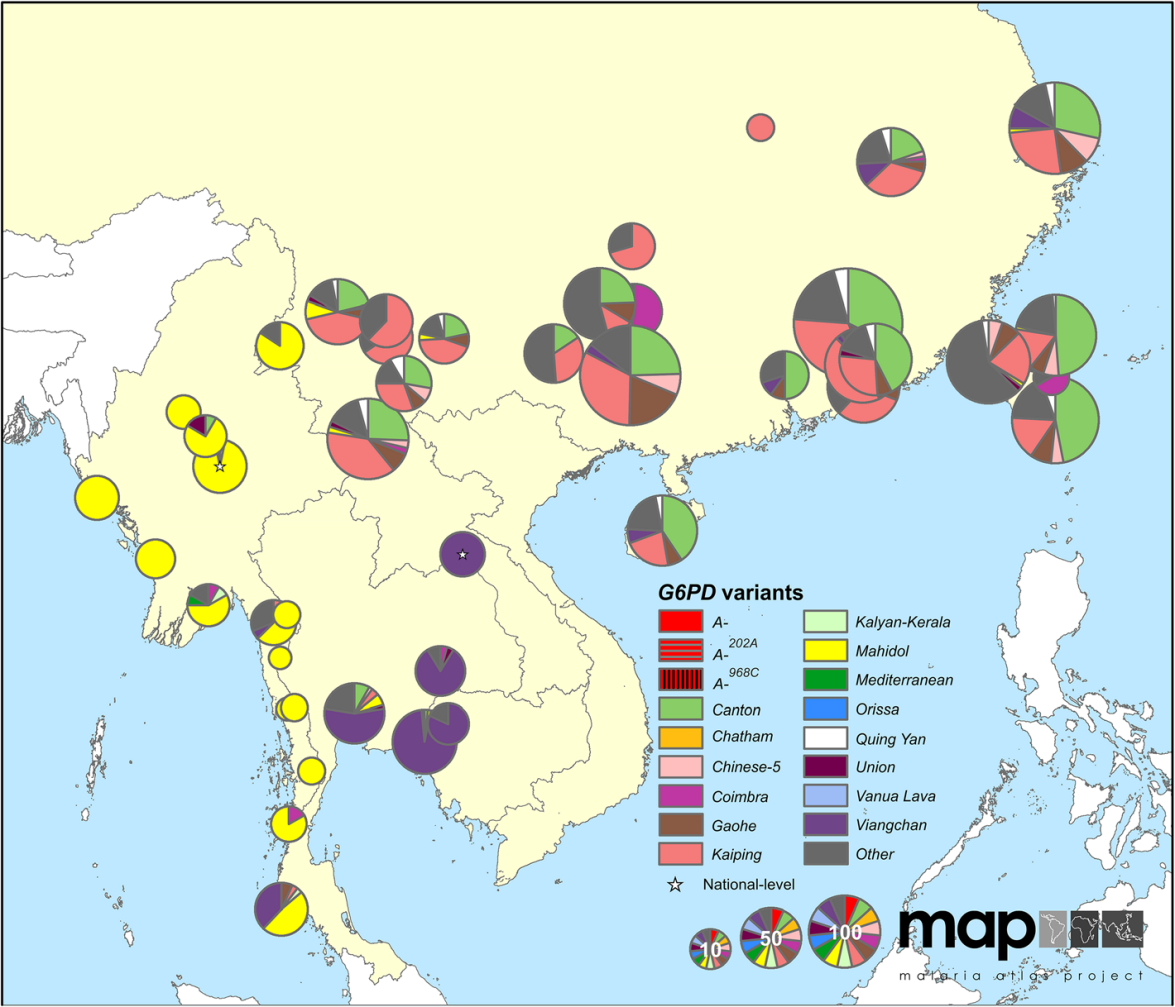
**G6PDd variant proportion map: East Asia (map series 1).** A higher resolution map of Figure 4.

**Figure 7:**
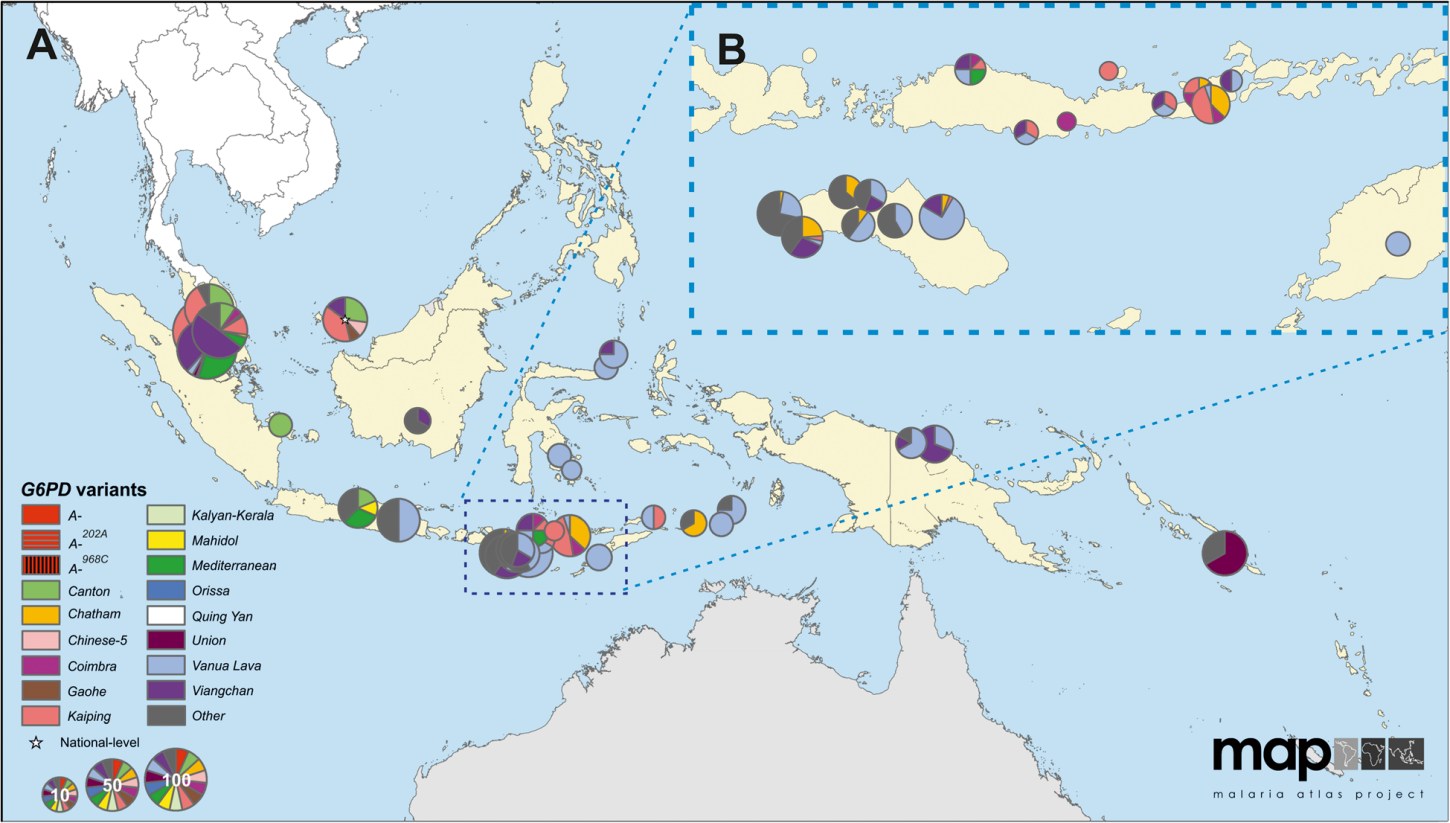
**G6PDd variant proportion map: Asia-Pacific (map series 1).** Panel **A** show the full map, which includes 36 surveys with a mean sample size of 18 (range: 1-128). One survey was mapped at the national level. The East Nusa Tenggara province islands identified by the dotted box are shown at higher resolution in Panel **B**. (See Figure 2 for full legend).

**Figure 8:**
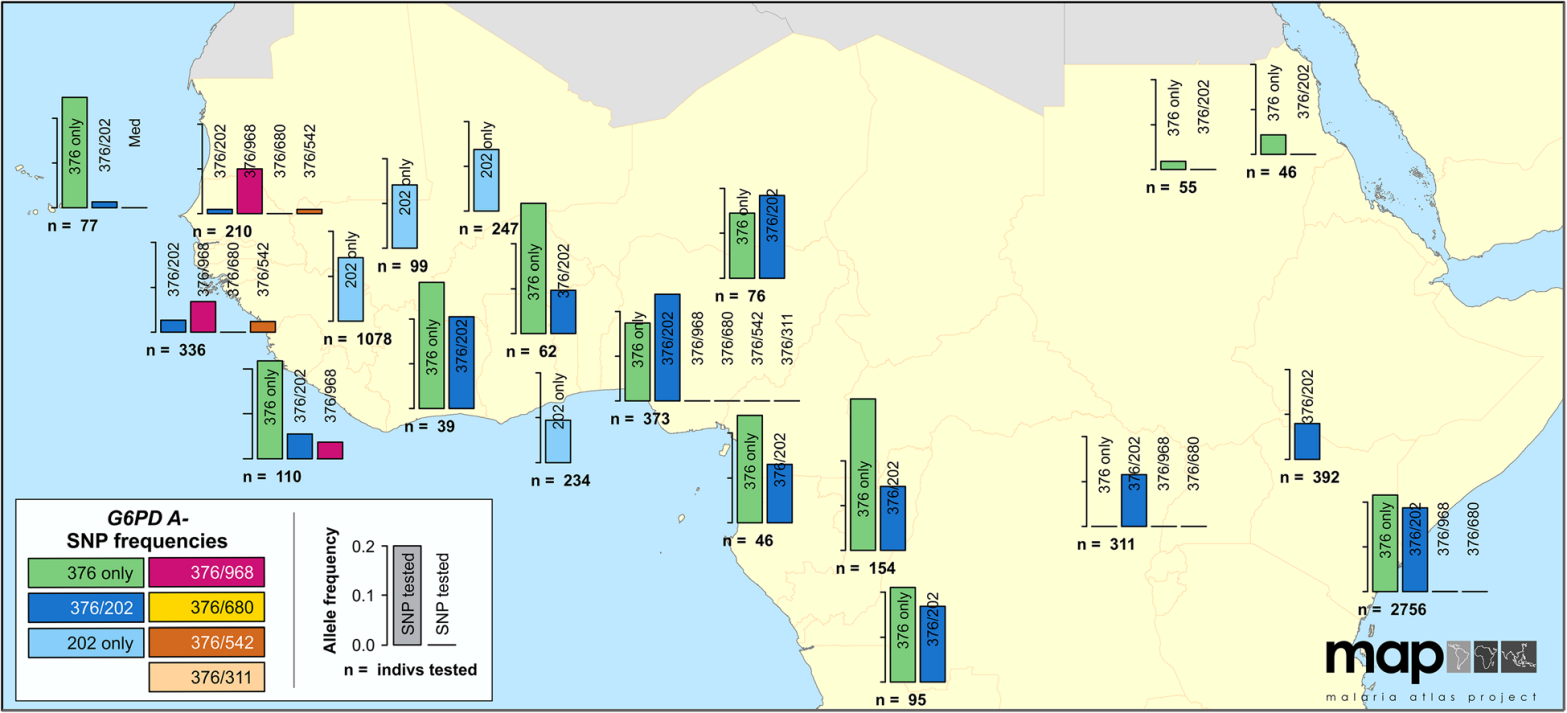
***G6PD A- *variant SNP frequency map for Africa (map series 2).** Bar charts representing the frequencies of *G6PD* variants. The variants that were tested for in each location are listed above the *x*-axis. Empty spaces along the *x*-axis indicate that the named variant was tested for but not identified in the population sample. Survey locations are mapped to closest approximation at the point of origin of the plots and exact locations shown in Additional file 4. Sample size is listed under each plot. Equivalent maps of the Americas and Asia are Additional files 3 and 5.

The maps reveal conspicuous distinct geographical patterns in the distribution and prevalence of *G6PD* variants across regions. The two series of maps represent both the relative proportions of the variants responsible for phenotypic G6PD enzyme deficiency (Figures 2, 3, 4, 5, 6, 7) and the allele frequencies of selected variants (Figure 8 and Additional files 3, 4 and 5). Together, these maps revealed a number of clear patterns further described below: (i) a relatively low diversity of *G6PD* variants reported from populations of the Americas and Africa + regions, among whom variants of the *G6PD A-* phenotype are predominantly searched for and reported; similarly, *G6PD Mediterranean* was predominant in west Asia (from Saudi Arabia and Turkey to India); (ii) a sharp shift in variants identified east of India, showing no admixture with the common variants of west Asia; (iii) high variant diversity in east Asia and the Asia-Pacific region, with multiple variants co-occurring and no single variant being predominant.

### *G6PD* variants in the Americas

Relatively few surveys were available from the Americas (nine in map series 1, one in map series 2). Figure 2A indicates that among G6PDd individuals, the predominant variant was *G6PD A-* (in the majority of cases encoded by the *G6PD A-*^*202A*^ mutation, though the *G6PD A-*^*968C*^ variant was common in some samples), identified in 91% of deficient individuals surveyed across the region (579 of 636 total G6PDd individuals surveyed). Other variants identified included *G6PD Mediterranean*^*563T*^ and *Seattle*^*844C*^ (the latter was classified as part of the “Other” category as it was only reported twice, both instances from Brazil where it reached a prevalence of 13% (n = 2/15, [Additional file 2: S63] and 5% (n = 9/196, [S30])). A survey of Mexicans reported 61% of deficient cases being due to various *G6PD A-* variants, but did not test for any other variant (e g, *G6PD Mediterranean*), explaining the large proportion of “Other” variants [Additional file 2: S85].

A single community screening survey could be identified which investigated *G6PD* variant allele frequencies. This was in Acrelândia, Brazil, and searched for only one single-nucleotide polymorphism (SNP): *G6PD A-*^*202A*^. An allele frequency of 6% (n = 509, [Additional file 2: S14]) was reported (see Additional file 3).

### *G6PD* variants in Africa, Yemen and Saudi Arabia (Africa+)

Very few studies (*n* = 6) investigating the variants of known G6PDd individuals were identified from the Africa + region (Figure 3). Where available, both in West Africa and the western Indian Ocean islands, surveys indicated *G6PD A-*^*202A*^ to be the predominant variant; in Saudi Arabia, the *G6PD Mediterranean* variant was responsible for 78% of G6PDd cases in two surveys in the western coastal city of Jeddah [Additional file 2: S6,S28].

Surveys investigating population-level frequencies of specific *G6PD* variants were more common (Figure 8 and Additional file 4). These consistently looked for SNPs of *G6PD A-*, notably focussed on the *G6PD A-*^*202*^ locus. Frequencies of this SNP were over 20% in surveys from Nigeria and Côte d’Ivoire in West Africa, and found at 19% in coastal Kenyan populations. The *G6PD A-*^*968C*^ mutation encodes a similar phenotype to *G6PD A-*^*202A*^ and was found to be the more common of the two in West African populations (in The Gambia and Senegal, for instance [Additional file 2: S21,S25]); despite this, only six of 20 surveys across Africa + included the *G6PD A-*^*968*^ SNP in their diagnoses. Large population surveys in East Africa (n = 311 in Uganda [Additional file 2: S38] and n = 2,756 in Kenya [Additional file 2: S76]) found no evidence of the *G6PD A-*^*968C*^ mutation despite frequencies of 12 and 19% of the *G6PD A-*^*202A*^ mutation, respectively.

Most population surveys (70%) in the Africa + region tested the *G6PD A-*^*376*^ locus. Although this mutation has barely any clinical effect, it is commonly co-inherited with mutations at other loci, together encoding the range of *G6PD A*- variants (Figure 8). The *G6PD A-*^*376G*^ variant had very high prevalence among sub-Saharan African populations, and in all cases affected over 10% of individuals surveyed; further, in a subset of ten out of 16 of these surveys, its frequency reached 25-50%. Exceptions to this high *G6PD A-*^*376G*^ prevalence were two surveys (n = 46 and 55) from northern Sudan, which reported low (<5%) prevalence [Additional file 2: S41]; the *G6PD A-*^*202A*^ mutation was not detected in these samples.

### *G6PD* variants in Asia and Asia-Pacific

Greatest *G6PD* variant diversity globally was across the Asia and Asia-Pacific regions (Figures 4, 5, 6, 7) where up to ten variants were reported to co-occur within single populations. Furthermore, significant proportions of “Other” cases were frequently reported in the variant proportion maps (map series 1), indicating that genetic diversity is greater than represented by the pie charts in these maps.

From Turkey to Pakistan (Figure 5), *G6PD Mediterranean* was predominant, identified in 728 of 895 G6PDd individuals examined (81%). Two variants, *G6PD Kalyan-Kerala*^*949A*^ and *Orissa*^*131G*^, were reported exclusively from Indian populations. On the Indian sub-continent, these two variants and the *G6PD Mediterranean* variant represented the majority (88% of n = 555 G6PDd individuals tested) of deficiency cases, though notable proportions (up to 80%) of “Other” cases were also reported from eastern and southern India.

East of India, a completely different set of variants emerged (Figure 6). *G6PD Mahidol*^*487A*^ predominated across Myanmar, with 98 of 117 G6PDd individuals (84%) diagnosed across 14 locations as carrying this variant. Variants common among G6PDd individuals in southeast China were largely unique to these populations. Of the 2,883 G6PDd cases from China, the commonly diagnosed variants included *G6PD Kaiping*^*1388A*^ (total G6PDd cases: 827; 29% across China), *Canton*^*1376T*^ (792 cases; 27%), *Gaohe*^*95G*^ (265 cases; 9%), *Chinese-5*^*1024T*^ (104 cases; 4%) and *Quing Yan*^*392T*^ (87 cases; 3%). The distribution of *G6PD Viangchan*^*871A*^ was diffuse, reportedly common from Laos (where examination of 15 G6PDd individuals all carried this variant [Additional file 2: S34]) and Cambodia (reported from 61 out of 64 G6PDd individuals [Additional file 2: S43]) to Papua New Guinea (where a sample of 13 G6PDd individuals included nine with this variant [Additional file 2: S33]).

Variants across the Asia-Pacific region were also highly heterogeneous (Figure 7). The common pattern emerging from the 36 surveys of deficient individuals identified from this region (n_indivs_ = 635) was the diversity of variants and the heterogeneity of their prevalence among different populations. Furthermore, it should be noted that 20 of these surveys tested fewer than ten G6PDd individuals, limiting the diversity that would be captured and thus the representativeness of these reports. The greatest diversity identified in a single survey was from the Malaysian neonatal screening programme in Kuala Lumpur, which recorded ten variants from an investigation of 86 G6PDd newborns [Additional file 2: S4]. The short spatial-scale heterogeneity of this co-occurring variant diversity is illustrated by a set of surveys across the East Nusa Tenggara province of Indonesia (Figure 7B). Amid this co-occurring variant heterogeneity, the *G6PD Vanua Lava*^*383C*^ variant was the most common variant reported from Indonesian studies (identified in 84 out of 249 G6PDd individuals tested).

Only three allele frequency surveys (map series 2) could be identified from the Asia region (of 24 globally) [Additional file 2: S66,S83]. *G6PD Mahidol* was most commonly tested for, with allele frequencies of 12% and 18% (n_tested_ = 353 and 11, respectively; Additional file 5). Two surveys in Thailand tested for a broader range of variants; one found five variants (*G6PD Canton, Kaiping, Mahidol, Union* and *Viangchan*; n_tested_ = 84) at allele frequencies of 1-2%; and the other, with a smaller sample size (n_tested_ = 11), identified only the *G6PD Mahidol* variant [Additional file 2: S66].

## Discussion

Over a third of the world’s population lives at risk of *P. vivax* infection [14,52,54]. Limited evidence underpins estimates of clinical cases, but these have been estimated at 70-400 million annually [55,56], including potentially severe illness and death [5,24,57,58]. In the context of malaria elimination, therapy must target all infections, including asymptomatic and submicroscopic blood-stage infections, dormant liver-stage hypnozoites as well as clinical cases [59,60]. One of the many consequences of a half-century of neglect of *P. vivax* has been the failure to address the primaquine toxicity problem with G6PDd and thereby solve the real problem of lack of access to primaquine for almost all malaria patients. No non-toxic therapeutic alternatives exist, and existing G6PDd diagnostics are largely impractical in point-of-care settings [4,6,21,45,56].

Fear of harm also leads to avoidance of primaquine as a *P. falciparum* transmission-blocking agent. The single transmission-blocking 0.75 mg/kg dose, consistently inconsequential in *G6PD A-* volunteers, reliably caused 20-30% drops in haematocrit in healthy Europeans with *G6PD Mediterranean*[39]. This risk, acknowledged at least 30 years ago, was only acted upon in 2012 with the WHO offering a recommendation for a lower dose of primaquine (0.25 mg/kg) gametocytocide alongside *P. falciparum* blood schizontocidal treatment with no requirement for prior G6PDd testing [10,11,61-63]. Nonetheless, many nations, in particular in Africa where there has been an almost complete lack of experience with primaquine [61] and where G6PDd prevalence is commonly greater than 10% [2], still resist use of this drug. An understanding of the therapeutic risks from primaquine in relation to the *G6PD* variants predominant in each region will contribute to the body of evidence on which to base policy.

Access to safe therapy either requires an alternative non-haemolytic drug or a practical means to identify G6PDd *P. vivax* malaria patients in remote, impoverished sites. Neither is likely in the short-term and improved use of primaquine promises immediately applicable and useful prospects [21]. This aim demands refined knowledge of the spatial epidemiology and primaquine-sensitivity phenotypes of *G6PD* variants. Development of primaquine treatment strategy could exploit, for example, the total dose effect (whereby therapeutic efficacy is dependent upon the total drug dose irrespective of the regimen duration [64-66]), to tailor population/region-specific dosing parameterized according to the highest dose safely tolerated by individuals with the most vulnerable local *G6PD* variant.

### *G6PD* variants in Africa

The G6PDd phenotype has long been considered homogenous across African populations, with much of the pre-molecular era literature reporting *G6PD A-* from electrophoresis assays across the continent [34]. The early investigations of “primaquine sensitivity” among *G6PD A-* volunteers found primaquine-induced haemolysis to be mild and self-limiting [1,30,64,67-69]. Nevertheless, the haemolytic susceptibility of this “mild” variant has been observed through severe reactions requiring transfusion [70], as well as through the failure of the Lapdap (chlorproguanil-dapsone) anti-malarial trials [71,72], and as haemolysis induced by the ingestion of fava beans [73], a pathology previously thought to only be triggered by more severe variants [74]. Haemolysis associated with *G6PD A-*, while perhaps less severe than with other variants in some circumstances or settings, ought not be universally characterized as “mild” as this risks leading to misplaced confidence in a relatively cavalier application of the drug.

Molecular analyses of *G6PD* variants among Africans revealed a diversity of SNPs within the umbrella of the *G6PD A-* phenotype [75]. The data, here mapped spatially, indicated a transition of SNP predominance from west (*G6PD A-*^*968C*^ polymorphism) to east (*G6PD A-*^*202A*^ widespread and *G6PD A-*^*968C*^ apparently absent). However, expansion of the relatively small number of surveys (n = 20) may reveal more complex distributions. Furthermore, the true diversity of *G6PD* variants cannot be discerned from the assembled surveys; it is not possible to know what proportion of genetic diversity is reflected in the map (Figure 3) because the diagnoses are limited to assessing the presence or absence of a limited number of anticipated SNPs, without any prior identification of phenotypically deficient hosts (as in map series 1). Full gene sequencing is the only reliable way of identifying all gene variants in the population. Genetic diversity within the umbrella *G6PD A-* phenotype may explain the spectrum of expression levels observed [70]: inheritance of a single SNP may not be sufficient to explain the observed phenotype. If these patterns of co-occurring SNP diversity encoding variable *G6PD A-* phenotypes are widely observed, a haplotype-based approach which allows for the co-inheritance of multiple SNPs may be required if the use of molecular diagnostics is to the scaled-up, as seems to be increasingly common [75]. Despite its obvious limitations to field-based applications, a gene-wide haplotype perspective would also allow increased compatibility with the rapidly expanding human genome sequencing efforts which are cataloguing human genetic diversity [76,77].

Although demand for primaquine has historically been low across Africa [78], it was Ethiopia that reported the highest number of *P. vivax* cases globally in 2011 (665,813 cases; [79]). G6PDd prevalence has been estimated at 1.0% in Ethiopia (50% CI: 0.7-1.5) [2], though no molecular information exists to indicate which variants may be responsible. Furthermore, beyond these areas with acknowledged endemic *P. vivax*, the status of transmission in human populations previously considered refractory to infection [78] is being brought into question by several lines of evidence [80-86]. The use of primaquine for *P. falciparum* control may also increase following the new WHO gametocytocide guidelines [63]. The elimination agenda includes African nations, with most located at the fringes of entrenched holo-endemic transmission [87,88], and the pressure to apply primaquine both as a hypnozoitocide for *P. vivax* and *Plasmodium ovale*, and as a gametocytocide for *P. falciparum* will increase with further progress towards zero transmission. A more detailed and targeted understanding of African *G6PD* epidemiology is necessary beyond the opportunistic surveys presented in this paper.

### *G6PD* variants in West Asia

The *G6PD Mediterranean* variant, appearing predominant across southwest Asia and exceedingly common in much of India (especially western regions), is known to be highly sensitive to AHA induced by primaquine. This wide distribution and dominance of *G6PD Mediterranean* presents a significant hurdle to the malaria elimination programmes in West Asia, which include Azerbaijan, Georgia, Iran, Iraq, Kyrgyzstan, Saudi Arabia, Tajikistan, Turkey, and Uzbekistan [89]. These nations must consider the relatively high risk of serious harm caused by unintentional dosing of G6PDd patients in need of malaria therapy. Implementation of G6PDd screening prior to primaquine radical cure therapy would greatly mitigate such risks and accelerate progress to malaria elimination in this region.

Although *G6PD Mediterranean* was common across Indian populations, two other variants were also frequently reported which were indigenous to populations from this subcontinent: *G6PD Kalyan-Kerala* and *Orissa*. Little is known of the primaquine sensitivity phenotypes of these two variants. Since almost half of the global population at risk of *P. vivax* malaria occurs in this single nation [52,79,90] studies characterizing such sensitivity would ultimately prove very useful in planning towards elimination.

### *G6PD* in East Asia and the West Pacific

The maps of eastern Asia and the western Pacific present the most complex picture of *G6PD* variants globally, coinciding with regions of heterogeneous and high *P. vivax* endemicity [52]. Almost all the common variants of public health concern globally were reported from this region. Reasons for this genetic diversity are unclear, but it is interesting that *P. falciparum* parasites (postulated to be selective agents of G6PDd [16]) have been found to show a greater degree of population structure with lower genetic relatedness between populations in Asia than across Africa [91]. As well as this overall diversity, the structure of *G6PD* variant heterogeneity with multiple variants co-occurring is starkly different from other areas where single variants predominate.

Despite large variant diversity across this region where many countries now target elimination (thus require primaquine therapy) [89], only one truly Asian variant has been examined in relation to AHA sensitivity to primaquine. *G6PD Mahidol*, common across Myanmar and parts of Thailand (Figure 6), exhibits residual G6PD enzyme activity ranging between 5-32% [42]. A handful of small primaquine sensitivity studies examining this variant have been conducted in Thailand, reporting very mild to moderate sensitivity to both 14-day and eight weekly primaquine regimens [40,92,93], as well as to single-dose regimens of *P. falciparum* transmission blocking therapy [94]. However, the *G6PD Mahidol* primaquine sensitivity phenotype must not be assumed to be representative of the region. As emphasized by the WHO Expert Review Group on primaquine in 2012 [62,63], far greater evidence-based understanding of these phenotypes among the many common Asian variants is urgently needed. The identity and distribution of variants as sensitive as *G6PD Mediterranean* in the Asia-Pacific region remains unexplored and unknown. This lack of understanding threatens patients and programmes with a more aggressive roll-out of primaquine therapy. In populations of such high heterogeneity, a dual approach of phenotypic screening followed by variant analysis of residual enzyme activity may be required to identify such risks.

The danger of under-diagnosing deficiency by using molecular identification alone (i e, the data informing map series 2) is illustrated by comparison of the two types of maps (Figure 2C and Additional file 5). If only a selected subset of perceived “common” variants are used in population screening surveys, a proportion of deficient individuals may be missed and put at risk from primaquine therapy: only those variants that are looked for can be identified. The maps presented here also repeatedly highlight considerable proportions of “Other” *G6PD* variants. These correspond to an unknown haemolytic risk, and hint towards an ever greater diversity of variants. Only full gene sequencing will allow full characterization of the diversity in this polymorphic gene.

## Conclusions

Both the failure to treat *P. vivax* infections and the treatment itself carry some risk of severe clinical complications [5,24,62] (Recht J, Ashley EA, White NJ:8-aminoquinolines safety review for WHO primaquine ERG, unpublished). Each repeated episode of acute *P. vivax* malaria carries possibilities of delayed or improper diagnosis, improper treatment, onward transmission, serious illness, and death [5,24]. Likewise, primaquine-induced AHA in G6PDd patients may provoke renal failure and require multiple transfusions for recovery [24,43]. Improper clinical management of primaquine-induced haemolysis may lead to death [24], although the relative risks of this remain poorly documented and are likely to be rare. Similarly, scaled-up application of low-dose transmission-blocking primaquine against *P. falciparum* will likely emerge as a key strategy towards elimination, especially in the context of artemisinin-resistant *P. falciparum* malaria. Evidence-based assessment of the risks incurred with primaquine therapy in any given region is essential for the development of rational strategies to minimize harm caused by either the parasite or the drug used to attack it. The maps presented in this article offer a first glimpse at the distribution of variants of public health significance across malaria-endemic regions. Their implications are discussed with respect to the regionally specific issues associated with the ramping up of primaquine therapy on the strategic road to elimination.

## Abbreviations

AHA: Acute haemolytic anaemia; CNSHA: Chronic non-spherocytic haemolytic anaemia; G6PDd: Glucose-6-phosphate dehydrogenase deficiency (phenotype); RBC: Red blood cell; SNP: Single nucleotide polymorphism; WHO: World Health Organization.

## Competing interests

The authors declare that they have no competing interests.

## Authors’ contributions

REH conceived the study and oversaw its design and implementation with guidance from FBP, JKB and SIH. REH wrote the first draft of the manuscript, and assembled the data with assistance from MD and WMM. AS, AWS and TNW contributed unpublished data; all authors participated in the interpretation of results and in the writing and editing of the manuscript. All authors read and approved the final manuscript.

## Supplementary Material

Additional file 1 Supplementary methods. Click here for file

Additional file 2 Sources from which the datapoints in the maps were identified. Click here for file

Additional file 3 **Map series 2: Americas.** Bar charts represent population surveys which examined the frequencies of selected *G6PD* variants in representative population samples. These population groups had not undergone prior G6PDd phenotype screening. The variants which were tested for in each location are listed above the *x*-axis. Survey locations are indicated by nearby black stars. Sample size is stated under each plot. Click here for file

Additional file 4 **Map series 2: Africa.** (A) Bar charts represent population surveys which examined the frequencies of selected *G6PD* variants in representative population samples. These population groups had not undergone prior G6PDd phenotype screening. The variants which were tested for in each location are listed above the *x*-axis. Sample size is stated under each plot. Survey locations are mapped to closest approximation at the point of origin of the plots; exact survey locations are shown in Panel B. (Panel A is reproduced from Figure 8 in the main manuscript). Click here for file

Additional file 5 **Map series 2: Asia.** Bar charts represent population surveys which examined the frequencies of selected *G6PD* variants in representative population samples. These population groups had not undergone prior G6PDd phenotype screening. The variants which were tested for in each location are listed above the *x*-axis. Survey locations are indicated by nearby black stars. Sample size is stated under each plot. Click here for file

## References

[B1] Carson PE Flanagan CL Ickes CE Alving AS Enzymatic deficiency in primaquine-sensitive erythrocytes Science 1956 124 484 485 13360274 10.1126/science.124.3220.484-a

[B2] Howes RE Piel FB Patil AP Nyangiri OA Gething PW Hogg MM Battle KE Padilla CD Baird JK Hay SI G6PD deficiency prevalence and estimates of affected populations in malaria endemic countries: a geostatistical model-based map PLoS Med 2012 9 e1001339 10.1371/journal.pmed.1001339 23152723 PMC3496665

[B3] The malERA Consultative Group on Diagnoses and Diagnostics A research agenda for malaria eradication: diagnoses and diagnostics PLoS Med 2011 8 e1000396 21311583 10.1371/journal.pmed.1000396 PMC3026696

[B4] Baird JK Surjadjaja C Consideration of ethics in primaquine therapy against malaria transmission Trends Parasitol 2011 27 11 16 10.1016/j.pt.2010.08.005 20846906

[B5] Baird JK Evidence and implications of mortality associated with acute *Plasmodium vivax* malaria Clin Microbiol Rev 2013 26 36 57 10.1128/CMR.00074-12 23297258 PMC3553673

[B6] Baird JK Reinventing primaquine for endemic malaria Expet Opin Emerg Drugs 2012 17 439 444 10.1517/14728214.2012.720252 PMC3514001 22998791

[B7] White NJ The role of anti-malarial drugs in eliminating malaria Malar J 2008 7 Suppl 1 S8 10.1186/1475-2875-7-S1-S8 19091042 PMC2604872

[B8] The malERA Consultative Group on Drugs A research agenda for malaria eradication: drugs PLoS Med 2011 8 e1000402 21311580 10.1371/journal.pmed.1000402 PMC3026688

[B9] White NJ Determinants of relapse periodicity in *Plasmodium vivax* malaria Malar J 2011 10 297 10.1186/1475-2875-10-297 21989376 PMC3228849

[B10] Bousema T Drakeley C Epidemiology and infectivity of *Plasmodium falciparum* and *Plasmodium vivax* gametocytes in relation to malaria control and elimination Clin Microbiol Rev 2011 24 377 410 10.1128/CMR.00051-10 21482730 PMC3122489

[B11] White NJ Primaquine to prevent transmission of falciparum malaria Lancet Infect Dis 2012 13 175 181 23182932 10.1016/S1473-3099(12)70198-6

[B12] WHO Guidelines for the treatment of malaria, second edition 2010 Geneva: World Health Organization

[B13] WHO Global plan for artemisinin resistance containment (GPARC) 2011 Geneva: WHO

[B14] White NJ Qiao LG Qi G Luzzatto L Rationale for recommending a lower dose of primaquine as a *Plasmodium falciparum* gametocytocide in populations where G6PD deficiency is common Malar J 2012 11 418 10.1186/1475-2875-11-418 23237606 PMC3546849

[B15] Maude RJ Socheat D Nguon C Saroth P Dara P Li G Song J Yeung S Dondorp AM Day NP White NJ White LJ Optimising strategies for *Plasmodium falciparum* malaria elimination in Cambodia: primaquine, mass drug administration and artemisinin resistance PLoS One 2012 7 e37166 10.1371/journal.pone.0037166 22662135 PMC3360685

[B16] Greene LS G6PD deficiency as protection against falciparum-malaria: an epidemiologic critique of population and experimental studies Yearb Phys Anthropol 1993 36 153 178 10.1002/ajpa.1330360609

[B17] Kwiatkowski DP How malaria has affected the human genome and what human genetics can teach us about malaria Am J Hum Genet 2005 77 171 192 10.1086/432519 16001361 PMC1224522

[B18] Beutler E G6PD: population genetics and clinical manifestations Blood Rev 1996 10 45 52 10.1016/S0268-960X(96)90019-3 8861278

[B19] Mueller I Galinski MR Baird JK Carlton JM Kochar DK Alonso PL del Portillo HA Key gaps in the knowledge of *Plasmodium vivax*, a neglected human malaria parasite Lancet Infect Dis 2009 9 555 566 10.1016/S1473-3099(09)70177-X 19695492

[B20] Cappellini MD Fiorelli G Glucose-6-phosphate dehydrogenase deficiency Lancet 2008 371 64 74 10.1016/S0140-6736(08)60073-2 18177777

[B21] Howes RE Battle KE Satyagraha AW Baird JK Hay SI G6PD deficiency: Global distribution, genetic variants and primaquine therapy Adv Parasitol 2013 81 133 201 23384623 10.1016/B978-0-12-407826-0.00004-7

[B22] Murray CJ Vos T Lozano R Naghavi M Flaxman AD Michaud C Ezzati M Shibuya K Salomon JA Abdalla S Aboyans V Abraham J Ackerman I Aggarwal R Ahn SY Ali MK Alvarado M Anderson HR Anderson LM Andrews KG Atkinson C Baddour LM Bahalim AN Barker-Collo S Barrero LH Bartels DH Basanez MG Baxter A Bell ML Benjamin EJ Disability-adjusted life years (DALYs) for 291 diseases and injuries in 21 regions, 1990-2010: a systematic analysis for the global burden of disease study 2010 Lancet 2012 380 2197 2223 10.1016/S0140-6736(12)61689-4 23245608

[B23] Lozano R Naghavi M Foreman K Lim S Shibuya K Aboyans V Abraham J Adair T Aggarwal R Ahn SY Alvarado M Anderson HR Anderson LM Andrews KG Atkinson C Baddour LM Barker-Collo S Bartels DH Bell ML Benjamin EJ Bennett D Bhalla K Bikbov B Bin Abdulhak A Birbeck G Blyth F Bolliger I Boufous S Bucello C Burch M Global and regional mortality from 235 causes of death for 20 age groups in 1990 and 2010: a systematic analysis for the global burden of disease study 2010 Lancet 2012 380 2095 2128 10.1016/S0140-6736(12)61728-0 23245604 PMC10790329

[B24] Lacerda MV Fragoso SC Alecrim MG Alexandre MA Magalhaes BM Siqueira AM Ferreira LC Araujo JR Mourao MP Ferrer M Castillo P Martin-Jaular L Fernandez-Becerra C del Portillo H Ordi J Alonso PL Bassat Q Postmortem characterization of patients with clinical diagnosis of *Plasmodium vivax* malaria: to what extent does this parasite kill? Clin Infect Dis 2012 55 e67 e74 10.1093/cid/cis615 22772803

[B25] Luzzatto L Orkin SH, Nathan DG, Ginsburg D, Look AT, Fisher DE, Lux SE Glucose-6-phosphate dehydrogenase deficiency Nathan and Oski’s Hematology of Infancy and Childhood 2009 7 Philadelphia: Saunders

[B26] Minucci A Moradkhani K Hwang MJ Zuppi C Giardina B Capoluongo E Glucose-6-phosphate dehydrogenase (G6PD) mutations database: review of the "old" and update of the new mutations Blood Cells Mol Dis 2012 48 154 165 10.1016/j.bcmd.2012.01.001 22293322

[B27] Mason PJ Vulliamy TJ Glucose-6-phosphate dehydrogenase (G6PD) deficiency: genetics Encyclopedia of Life Sciences 2005 Chichester: John Wiley & Sons, Ltd

[B28] WHO Working Group Glucose-6-phosphate dehydrogenase deficiency Bull World Health Organ 1989 67 601 611 2633878 PMC2491315

[B29] Yoshida A Beutler E Motulsky AG Human glucose-6-phosphate dehydrogenase variants Bull World Health Organ 1971 45 243 253 5316621 PMC2427914

[B30] Hockwald RS Arnold J Clayman CB Alving AS Toxicity of primaquine in Negroes JAMA 1952 149 1568 1570 10.1001/jama.1952.72930340027010c 14945981

[B31] Beutler E Glucose-6-phosphate dehydrogenase deficiency N Engl J Med 1991 324 169 174 10.1056/NEJM199101173240306 1984194

[B32] Dern RJ Beutler E Alving AS The hemolytic effect of primaquine. II. The natural course of the hemolytic anemia and the mechanism of its self-limited character J Lab Clin Med 1954 44 171 176 13184224

[B33] Alving AS Johnson CF Tarlov AR Brewer GJ Kellermeyer RW Carson PE Mitigation of the haemolytic effect of primaquine and enhancement of its action against exoerythrocytic forms of the Chesson strain of *Plasmodium vivax* by intermittent regimens of drug administration: a preliminary report Bull World Health Organ 1960 22 621 631 13793053 PMC2555355

[B34] Cavalli-Sforza LL Menozzi P Piazza A The History and Geography of Human Genes 1994 Princeton, New Jersey: Princeton University Press

[B35] Piomelli S Corash LM Davenport DD Miraglia J Amorosi EL In vivo lability of glucose-6-phosphate dehydrogenase in Gd^A-^ and Gd^Mediterranean^ deficiency J Clin Invest 1968 47 940 948 10.1172/JCI105786 5641629 PMC297242

[B36] Meloni T Forteleoni G Dore A Cutillo S Favism and hemolytic anemia in glucose-6-phosphate dehydrogenase-deficient subjects in North Sardinia Acta Haematol 1983 70 83 90 10.1159/000206700 6408883

[B37] Luisada A Favism. JAMA 1940 115 632 632

[B38] Beutler E Duparc S Glucose-6-phosphate dehydrogenase deficiency and antimalarial drug development Am J Trop Med Hyg 2007 77 779 789 17978087

[B39] Clyde DF Clinical problems associated with the use of primaquine as a tissue schizontocidal and gametocytocidal drug Bull World Health Organ 1981 59 391 395 6976846 PMC2396064

[B40] Buchachart K Krudsood S Singhasivanon P Treeprasertsuk S Phophak N Srivilairit S Chalermrut K Rattanapong Y Supeeranuntha L Wilairatana P Brittenham G Looareesuwan S Effect of primaquine standard dose (15 mg/day for 14 days) in the treatment of vivax malaria patients in Thailand Southeast Asian J Trop Med Public Health 2001 32 720 726 12041544

[B41] Matsuoka H Wang J Hirai M Arai M Yoshida S Kobayashi T Jalloh A Lin K Kawamoto F Glucose-6-phosphate dehydrogenase (G6PD) mutations in Myanmar: G6PD Mahidol (487G > A) is the most common variant in the Myanmar population J Hum Genet 2004 49 544 547 10.1007/s10038-004-0187-7 15349799

[B42] Louicharoen C Patin E Paul R Nuchprayoon I Witoonpanich B Peerapittayamongkol C Casademont I Sura T Laird NM Singhasivanon P Quintana-Murci L Sakuntabhai A Positively selected G6PD-Mahidol mutation reduces *Plasmodium vivax* density in Southeast Asians Science 2009 326 1546 1549 10.1126/science.1178849 20007901

[B43] Burgoine KL Bancone G Nosten F The reality of using primaquine Malar J 2010 9 376 10.1186/1475-2875-9-376 21184691 PMC3018394

[B44] Laosombat V Sattayasevana B Chotsampancharoen T Wongchanchailert M Glucose-6-phosphate dehydrogenase variants associated with favism in Thai children Int J Hematol 2006 83 139 143 10.1532/IJH97.A20513 16513531

[B45] Baird JK Elimination therapy for the endemic malarias Curr Infect Dis Rep 2012 14 227 237 10.1007/s11908-012-0250-z 22415581 PMC3342493

[B46] Mason PJ Bautista JM Gilsanz F G6PD deficiency: the genotype-phenotype association Blood Rev 2007 21 267 283 10.1016/j.blre.2007.05.002 17611006

[B47] Mourant AE Kopec AC Domaniewska-Sobczak K The distribution of the human blood groups and other polymorphisms 1976 London: Oxford University Press

[B48] Livingstone FB Frequencies of hemoglobin variants: thalassemia, the glucose-6-phosphate dehydrogenase deficiency, G6PD variants and ovalocytosis in human populations 1985 New York: Oxford University Press

[B49] Nkhoma ET Poole C Vannappagari V Hall SA Beutler E The global prevalence of glucose-6-phosphate dehydrogenase deficiency: a systematic review and meta-analysis Blood Cells Mol Dis 2009 42 267 278 10.1016/j.bcmd.2008.12.005 19233695

[B50] Singh S Distribution of certain polymorphic traits in populations of the Indian peninsula and South Asia Isr J Med Sci 1973 9 1225 1237 4359640

[B51] Gething PW Patil AP Smith DL Guerra CA Elyazar IR Johnston GL Tatem AJ Hay SI A new world malaria map: *Plasmodium falciparum* endemicity in 2010 Malar J 2011 10 378 10.1186/1475-2875-10-378 22185615 PMC3274487

[B52] Gething PW Elyazar IR Moyes CL Smith DL Battle KE Guerra CA Patil AP Tatem AJ Howes RE Myers MF George DB Horby P Wertheim HFL Price RN Mueller I Baird JK Hay SI A long neglected world malaria map: *Plasmodium vivax* endemicity in 2010 PLoS Negl Trop Dis 2012 6 e1814 10.1371/journal.pntd.0001814 22970336 PMC3435256

[B53] Hay SI Guerra CA Gething PW Patil AP Tatem AJ Noor AM Kabaria CW Manh BH Elyazar IR Brooker S Smith DL Moyeed RA Snow RW A world malaria map: *Plasmodium falciparum* endemicity in 2007 PLoS Med 2009 6 e1000048 19323591 10.1371/journal.pmed.1000048 PMC2659708

[B54] Battle KE Gething PW Elyazar IR Moyes CL Sinka ME Howes RE Guerra CA Price RN Baird KJ Hay SI The global public health significance of *Plasmodium vivax* Adv Parasitol 2012 80 1 111 23199486 10.1016/B978-0-12-397900-1.00001-3

[B55] Hay SI Guerra CA Tatem AJ Noor AM Snow RW The global distribution and population at risk of malaria: past, present, and future Lancet Infect Dis 2004 4 327 336 10.1016/S1473-3099(04)01043-6 15172341 PMC3145123

[B56] Mendis K Sina BJ Marchesini P Carter R The neglected burden of *Plasmodium vivax* malaria Am J Trop Med Hyg 2001 64 97 106 11425182 10.4269/ajtmh.2001.64.97

[B57] Singh H Parakh A Basu S Rath B *Plasmodium vivax* malaria: is it actually benign? J Infect Pub Health 2011 4 91 95 10.1016/j.jiph.2011.03.002 21663878

[B58] Mahgoub H Gasim GI Musa IR Adam I Severe *Plasmodium vivax* malaria among Sudanese children at New Halfa Hospital, Eastern Sudan Parasit Vectors 2012 5 154 10.1186/1756-3305-5-154 22846165 PMC3464670

[B59] Douglas NM Nosten F Ashley EA Phaiphun L van Vugt M Singhasivanon P White NJ Price RN *Plasmodium vivax* recurrence following falciparum and mixed species malaria: risk factors and effect of antimalarial kinetics Clin Infect Dis 2011 52 612 620 10.1093/cid/ciq249 21292666 PMC3060895

[B60] Harris I Sharrock WW Bain LM Gray KA Bobogare A Boaz L Lilley K Krause D Vallely A Johnson ML Gatton ML Shanks GD Cheng Q A large proportion of asymptomatic *Plasmodium* infections with low and sub-microscopic parasite densities in the low transmission setting of Temotu Province, Solomon Islands: challenges for malaria diagnostics in an elimination setting Malar J 2010 9 254 10.1186/1475-2875-9-254 20822506 PMC2944341

[B61] Eziefula AC Gosling R Hwang J Hsiang MS Bousema T Von Seidlein L Drakeley C On behalf of the Primaquine in Africa Discussion Group Rationale for short course primaquine in Africa to interrupt malaria transmission Malar J 2012 11 360 10.1186/1475-2875-11-360 23130957 PMC3502539

[B62] Malaria Policy Advisory Committee Meeting WHO Evidence Review Group Report: the safety and effectiveness of single dose primaquine as a P. falciparum gametocytocide 2012 Geneva: World Health Organization

[B63] Global Malaria Programme WHO Updated WHO Policy Recommendation: Single dose Primaquine as a gametocytocide in Plasmodium falciparum malaria 2012 Geneva: World Health Organization

[B64] Edgcomb JH Arnold J Yount EH Jr Alving AS Eichelberger L Jeffery GM Eyles D Young MD Primaquine, SN 13272, a new curative agent in vivax malaria; a preliminary report J Natl Malar Soc 1950 9 285 292 14804087

[B65] Alving AS Hankey DD Coatney GR Jones R Jr Coker WG Garrison PL Donovan WN Korean vivax malaria. II. Curative treatment with pamaquine and primaquine Am J Trop Med Hyg 1953 2 970 976 13104805

[B66] Coatney GR Alving AS Jones R Jr Hankey DD Robinson DH Garrison PL Coker WG Donovan WN Di Lorenzo A Marx RL Simmons IH Korean vivax malaria. V. Cure of the infection by primaquine administered during long-term latency Am J Trop Med Hyg 1953 2 985 988 13104808

[B67] Flanagan CL Schrier SL Carson PE Alving AS The hemolytic effect of primaquine. VIII. The effect of drug administration on parameters of primaquine sensitivity J Lab Clin Med 1958 51 600 608 13525836

[B68] Beutler E The hemolytic effect of primaquine and related compounds: a review Blood 1959 14 103 139 13618370

[B69] Alving AS Craige B Pullman TN Whorton CM Jones R Eichelberger L Procedures used at Stateville Penitentiary for the testing of potential antimalarial agents J Clin Invest 1948 27 2 5 10.1172/JCI101956 PMC438882 16695630

[B70] Shekalaghe SA ter Braak R Daou M Kavishe R van den Bijllaardt W van den Bosch S Koenderink JB Luty AJ Whitty CJ Drakeley C Sauerwein RW Bousema T In Tanzania, hemolysis after a single dose of primaquine coadministered with an artemisinin is not restricted to glucose-6-phosphate dehydrogenase-deficient (G6PD A-) individuals Antimicrob Agents Chemother 2010 54 1762 1768 10.1128/AAC.01135-09 20194698 PMC2863610

[B71] Luzzatto L The rise and fall of the antimalarial Lapdap: a lesson in pharmacogenetics Lancet 2010 376 739 741 10.1016/S0140-6736(10)60396-0 20599264

[B72] Pamba A Richardson ND Carter N Duparc S Premji Z Tiono AB Luzzatto L Clinical spectrum and severity of hemolytic anemia in glucose 6-phosphate dehydrogenase-deficient children receiving dapsone Blood 2012 120 4123 4133 10.1182/blood-2012-03-416032 22993389

[B73] Galiano S Gaetani GF Barabino A Cottafava F Zeitlin H Town M Luzzatto L Favism in the African type of glucose-6-phosphate dehydrogenase deficiency (A-) BMJ 1990 300 236 2106932 10.1136/bmj.300.6719.236 PMC1662078

[B74] Mehta AB Glucose-6-phosphate dehydrogenase deficiency Postgrad Med J 1994 70 871 877 10.1136/pgmj.70.830.871 7870632 PMC2398001

[B75] Clark TG Fry AE Auburn S Campino S Diakite M Green A Richardson A Teo YY Small K Wilson J Jallow M Sisay-Joof F Pinder M Sabeti P Kwiatkowski DP Rockett KA Allelic heterogeneity of G6PD deficiency in West Africa and severe malaria susceptibility Eur J Hum Genet 2009 17 1080 1085 10.1038/ejhg.2009.8 19223928 PMC2986558

[B76] 1000 Genomes - A Deep Catalog of Human Genetic Variation http://www.1000genomes.org

[B77] International HapMap Project http://hapmap.ncbi.nlm.nih.gov

[B78] Howes RE Patil AP Piel FB Nyangiri OA Kabaria CW Gething PW Zimmerman PA Barnadas C Beall CM Gebremedhin A Menard D Williams TN Weatherall DJ Hay SI The global distribution of the Duffy blood group Nat Commun 2011 2 266 21468018 10.1038/ncomms1265 PMC3074097

[B79] WHO World Malaria Report 2012 2012 Geneva: World Health Organization

[B80] Boyd MF An Introduction to Malariology 1930 Cambridge, MA: Harvard University Press

[B81] Guerra CA Howes RE Patil AP Gething PW Van Boeckel TP Temperley WH Kabaria CW Tatem AJ Manh BH Elyazar IR Baird JK Snow RW Hay SI The international limits and population at risk of *Plasmodium vivax* transmission in 2009 PLoS Negl Trop Dis 2010 4 e774 10.1371/journal.pntd.0000774 20689816 PMC2914753

[B82] Ryan JR Stoute JA Amon J Dunton RF Mtalib R Koros J Owour B Luckhart S Wirtz RA Barnwell JW Rosenberg R Evidence for transmission of *Plasmodium vivax* among a duffy antigen negative population in Western Kenya Am J Trop Med Hyg 2006 75 575 581 17038676

[B83] Culleton R Ndounga M Zeyrek FY Coban C Casimiro PN Takeo S Tsuboi T Yadava A Carter R Tanabe K Evidence for the transmission of *Plasmodium vivax* in the Republic of the Congo, West Central Africa J Infect Dis 2009 200 1465 1469 10.1086/644510 19803728

[B84] Menard D Barnadas C Bouchier C Henry-Halldin C Gray LR Ratsimbasoa A Thonier V Carod JF Domarle O Colin Y Bertrand O Picot J King CL Grimberg BT Mercereau-Puijalon O Zimmerman PA *Plasmodium vivax* clinical malaria is commonly observed in duffy-negative malagasy people Proc Natl Acad Sci USA 2010 107 5967 5971 10.1073/pnas.0912496107 20231434 PMC2851935

[B85] Mendes C Dias F Figueiredo J Mora VG Cano J De Sousa B Do Rosario VE Benito A Berzosa P Arez AP Duffy negative antigen is no longer a barrier to *plasmodium vivax*--molecular evidences from the African West Coast (Angola and Equatorial Guinea) PLoS Negl Trop Dis 2011 5 e1192 10.1371/journal.pntd.0001192 21713024 PMC3119644

[B86] Wurtz N Mint Lekweiry K Bogreau H Pradines B Rogier C Ould Mohamed Salem Boukhary A Hafid JE Ould Ahmedou Salem MS Trape JF Basco LK Briolant S Vivax malaria in mauritania includes infection of a duffy-negative individual Malar J 2011 10 336 10.1186/1475-2875-10-336 22050867 PMC3228859

[B87] Feachem RG Phillips AA Hwang J Cotter C Wielgosz B Greenwood BM Sabot O Rodriguez MH Abeyasinghe RR Ghebreyesus TA Snow RW Shrinking the malaria map: progress and prospects Lancet 2010 376 1566 1578 10.1016/S0140-6736(10)61270-6 21035842 PMC3044848

[B88] Cotter C Sturrock HJ Hsiang MS Liu J Phillips AA Hwang J Gueye CS Fullman N Gosling RD Feachem RG The changing epidemiology of malaria elimination: new strategies for new challenges Lancet 2013 382 9895 900 911 10.1016/S0140-6736(13)60310-4 23594387 PMC10583787

[B89] UCSF Global Health Group and Malaria Atlas Project Atlas of Malaria-Eliminating Countries 2011 University of California, San Francisco: The Global Health Group, Global Health Sciences

[B90] Hay SI Gething PW Snow RW India’s invisible malaria burden Lancet 2010 376 1716 1717 10.1016/S0140-6736(10)61084-7 20970180 PMC3039297

[B91] Manske M Miotto O Campino S Auburn S Almagro-Garcia J Maslen G O’Brien J Djimde A Doumbo O Zongo I Ouedraogo JB Michon P Mueller I Siba P Nzila A Borrmann S Kiara SM Marsh K Jiang H Su XZ Amaratunga C Fairhurst R Socheat D Nosten F Imwong M White NJ Sanders M Anastasi E Alcock D Drury E Analysis of *Plasmodium falciparum* diversity in natural infections by deep sequencing Nature 2012 487 375 379 10.1038/nature11174 22722859 PMC3738909

[B92] Takeuchi R Lawpoolsri S Imwong M Kobayashi J Kaewkungwal J Pukrittayakamee S Puangsa-art S Thanyavanich N Maneeboonyang W Day NP Singhasivanon P Directly-observed therapy (DOT) for the radical 14-day primaquine treatment of *Plasmodium vivax* malaria on the Thai-Myanmar border Malar J 2010 9 308 10.1186/1475-2875-9-308 21040545 PMC2988041

[B93] Myat Phone K Myint O Aung N Aye Lwin H The use of primaquine in malaria infected patients with red cell glucose-6-phosphate dehydrogenase (G6PD) deficiency in Myanmar Southeast Asian J Trop Med Public Health 1994 25 710 713 7667719

[B94] Song J Socheat D Tan B Dara P Deng C Sokunthea S Seila S Ou F Jian H Li G Rapid and effective malaria control in Cambodia through mass administration of artemisinin-piperaquine Malar J 2010 9 57 10.1186/1475-2875-9-57 20175930 PMC2837675

