# Supplementary material for: Spatial distribution of G6PD deficiency variants across malaria-endemic regions
Source: Malar J. 2013 Nov 15;12:418. doi: 10.1186/1475-2875-12-418 (PMC3835423; doi:10.1186/1475-2875-12-418)
Supplement: Additional file 1 — Supplementary methods. [file 1475-2875-12-418-S1.docx]

**Spatial distribution of G6PD deficiency variants across malaria endemic regions**

Additional Protocol 1 – Supplementary Methods

This supplementary section describes in more detail some aspects of the methods used in this study.

Library assembly

The first methodological step was a literature search to identify sources of representative population surveys of G6PDd, using the protocol previously described [[1](#_ENREF_1)]. In summary, these used systematic keyword searches (“G6PD”, “glucose-6-phosphate dehydrogenase” or “glucose 6 phosphate dehydrogenase”) of major online biomedical literature databases (PubMed, Web of Knowledge and Scopus; last conducted systematically on 20 March 2013) and cross-checks with existing databases [[2-7](#_ENREF_2)]. Requests for unpublished data sets were also made to researchers active in the field.

Survey selection criteria

Three initial inclusion criteria were imposed:

1. Spatial specificity: only population surveys which could be geopositioned to at least the national level were included. Surveys were mapped to the highest resolution spatial scale available, ideally as point locations (e.g. villages).
2. Community representativeness: to ensure that population samples were representative of the communities being surveyed, only studies which provided unbiased prevalence estimates were included. Case studies or other patient groups, particularly those with symptoms of severe G6PDd (such as hyperbilirubinaemia, kernicterus or kidney failure), were excluded on account of being more likely to include individuals with severe variants. Malaria patients were excluded due to a potential advantage conferred by G6PDd, which would underestimate frequencies of the most protective variants; furthermore, if – hypothetically – those variants which confer the strongest protective effect are the most clinically severe, these surveys would have dangerously misrepresentative implications for mass applications of potentially haemolytic drugs. Family studies were excluded for being unrepresentative of the wider community due to their high degree of consanguinity. Finally, studies which included only individuals of selected ethnic backgrounds in mixed populations were also excluded to ensure that the data collated would be widely representative.
3. Molecular diagnosis: to exclude the diagnostic uncertainty from surveys reliant on biochemical diagnoses in difficult field conditions, only surveys employing molecular methodologies were included. This is further discussed below.

Molecular vs. biochemical G6PDd diagnosis

*G6PD* variants have historically been diagnosed both with biochemical and genetic methods (Figure A1): either the enzyme itself must be thoroughly characterised with a suite of biochemical investigations of the purified enzyme, or genetic analysis must identify the underlying mutations. However, the highly involved and demanding nature of the laboratory analyses required for biochemical variant characterisation (including enzyme kinetics, electrophoretic mobility, heat stability, activity-pH curves and Michaelis constant measurements [[8](#_ENREF_8)]) means that they are rarely feasible in field settings to the recommended standards [[8](#_ENREF_8), [9](#_ENREF_9)], especially in older surveys when sophisticated laboratory equipment was less widely available and the confounding effect of protein degradation harder to control. Complexity is also introduced into biochemical variant diagnoses by variations in kinetic measurements even within samples of the same variant. In general, therefore, reports of biochemical diagnoses are rarely as reliable as the direct evidence from examination of mutations in the *G6PD* gene’s DNA. In this study, therefore, only surveys which used molecular diagnostics were included for mapping.


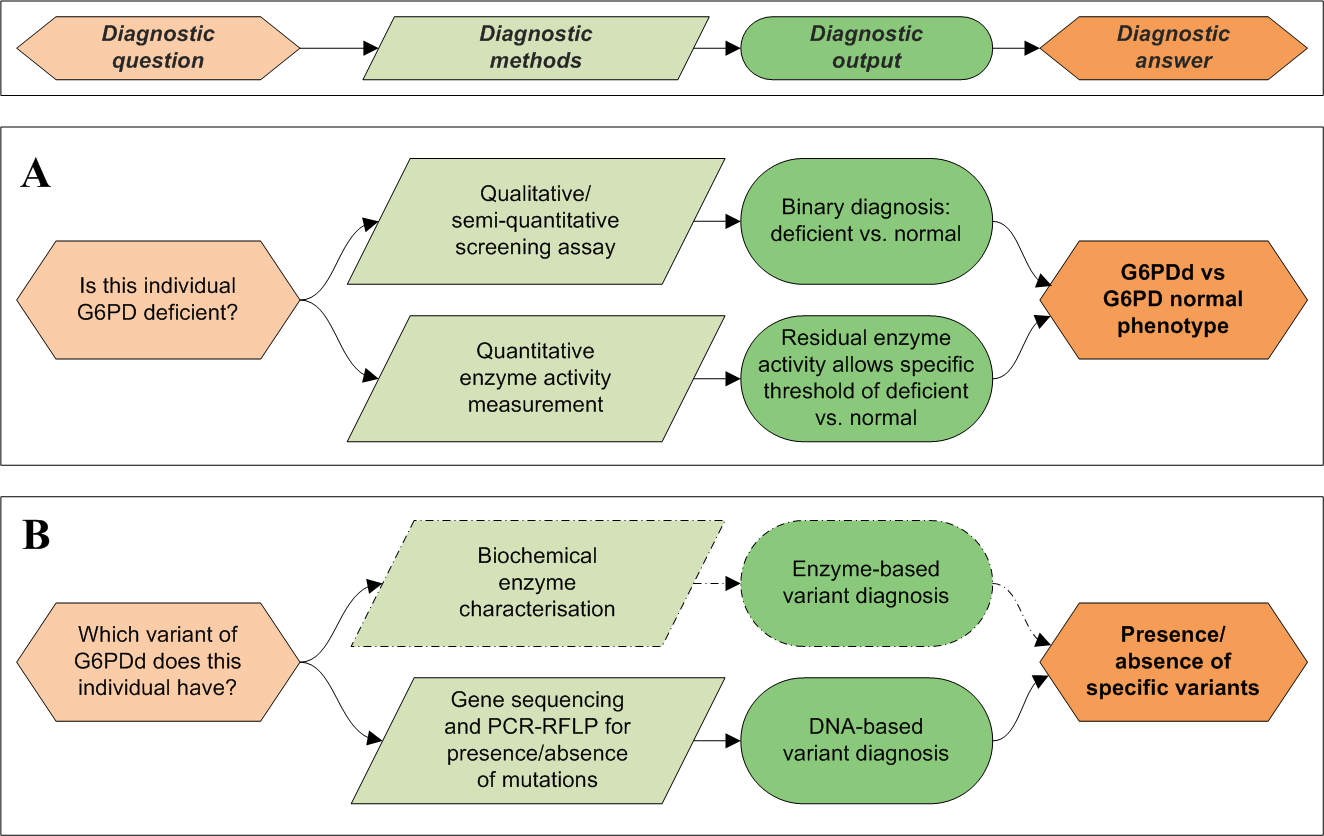


**Figure A1. G6PDd diagnostic methods and common laboratory techniques associated with different types of diagnostic questions**. Panel A summarises diagnostics related to identifying deficient from normal G6PD enzyme activity. Panel B indicates the methods required to characterise the variants of G6PDd. The orange hexagons indicate the question and answers associated with the different methods. The different diagnostic methods associated with each are shown in the pale green boxes, and the diagnostic outcomes of each are shown in the bright green ellipses.

Variant inclusion criteria

Given the genetic diversity of *G6PD* variants, it was necessary to identify those variants which presented a prominent public health threat. The study focused on Type 2 *G6PD* variants. As detailed in Table 1, these variants:

- have significantly reduced residual enzyme activity (<50% normal expression) and are thus diagnosable as deficient by standard qualitative diagnostics (Figure A1),
- are associated with significant clinical symptoms by predisposing individuals to acute haemolytic anaemia triggered by food or drugs
- reach polymorphic frequencies (>1% prevalence) and are therefore relatively common in affected communities.

For clarity, not all *G6PD* variants could be included in the maps, so a minimum reporting threshold was imposed. Variants reported from at least ten localities across the malaria endemic region were included (Figure A2). Across the database, 15 variants met these criteria and were included in the mapping.


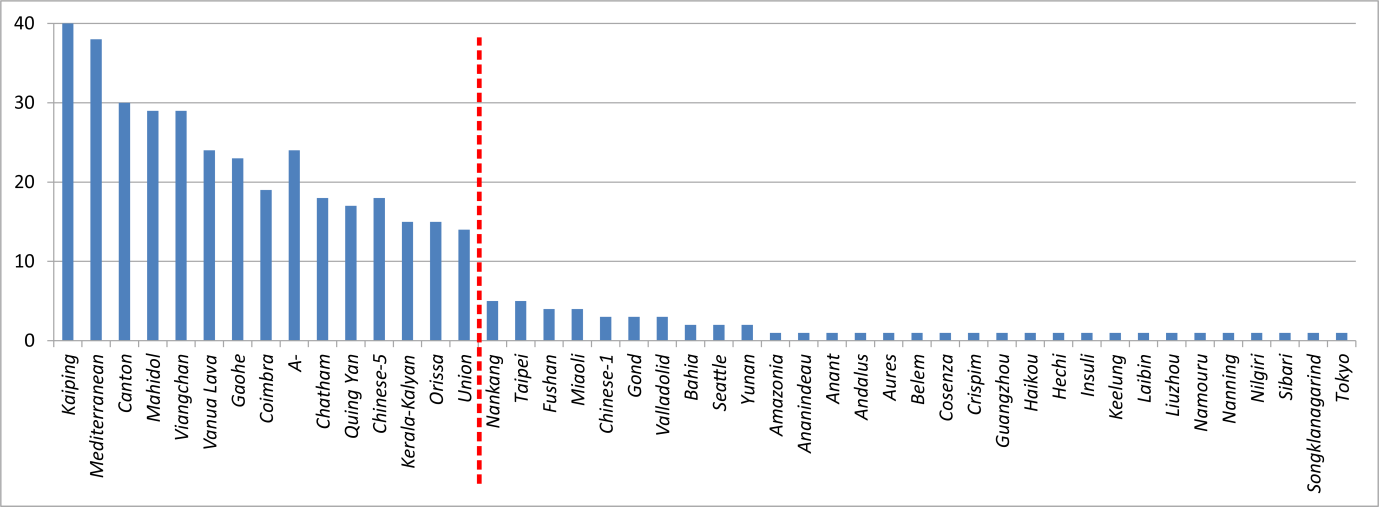


**Figure A2. Occurrences of each *G6PD* variant in the assembled database of** **surveys**. Variants to the left of the red dotted line were reported from more than 10 locations and were included in the maps.

The *G6PD A-* variant is an overarching phenotype which encompasses several single nucleotide polymorphisms (SNPs), usually inherited alongside the A376G mutation (thus inherited as G202A/A376G or T968C/A376G or G680T/A376G or “Santamaria” A542T/A376G). Where sufficient detail was available, occurrences of the *G6PD A-* variant were recorded in the database by SNP.

Variable variant nomenclature was standardised according to the mutations encoding the deficiency: for example, the *G6PD^G871A^* mutation is common to both *G6PD Viangchan* and *G6PD Jammu* variants, although these are distinguished by haplotype analysis of a non-coding locus which is not frequently examined [[10](#_ENREF_10)]; these two variants were therefore considered a single variant in this study.

Mapping the data

Surveys satisfying the inclusion criteria were abstracted into a database and mapped spatially. The malaria endemicity limits were those previously described [[1](#_ENREF_1), [11](#_ENREF_11), [12](#_ENREF_12)], corresponding to 99 *P. vivax* and *P. falciparum* endemic countries in 2010. The geographic regions used were selected for consistency with previous Malaria Atlas Project subdivisions, based on malaria epidemiological characteristics [[11](#_ENREF_11), [12](#_ENREF_12)]. These are: Americas, Africa+ (Africa, Saudi Arabia, Yemen), Asia (subdivided into West and East Central Asia, and the Pacific region; as shown in Figures 4-7). All mapping was performed in ArcMap 10 (ESRI, Redlands, CA, USA). The two types of data were mapped into variant proportion maps and variant allele frequency maps.

1. Map series 1: Variant proportion maps

Pie charts were used to display the variant proportion data. These represented the relative proportions of each variant in the sample of G6PDd individuals examined (irrespective of gender), without providing any estimate of their overall population-level frequencies; their denominator was the number of G6PDd individuals in the study. Confidence in the data, as represented by sample size, was incorporated through pie chart size, with larger pie charts representing bigger sample sizes. These had to be transformed on a square-root scale to allow their clear visualisation in a single map due to the large range of sample sizes. Surveys which could only be mapped to the national level were indicated by a white star in the centre of the pie charts; pie charts without stars were therefore mapped with greater precision, from the village- to province-level. Spatial duplicates from independent studies, where multiple surveys had been conducted among the same communities, were mapped with a “jitter” of 0.5-1° in their latitude or longitude decimal degree coordinates to allow visualisation of multiple charts for the same location.

1. Map series 2: Variant frequency maps

Surveys which investigated *G6PD* variants in individuals from cross-sectional population samples with no prior G6PDd screening were included in map series 2. These studies estimated the allele frequencies of selected variants at the population level, and were mapped spatially using bar charts to represent the allele frequencies. This visualisation conveyed the important concept that frequency estimates could only be available for variants which were included in the diagnoses, underlying the importance of knowing which variants were included in the diagnostic analyses when interpreting their significance. The variants tested for in the studies were represented along the bars of the $x$-axis of the graphs. Empty spaces along the $x$-axis indicate that the named variant was tested for but not identified from the population sample.

Accurate placement of the bar charts on a map was more difficult than the pie charts. Their true locations were mapped with black stars (see Additional Files 3-5).

Given that the *G6PD* gene is X-linked, deriving estimates of allele frequency required the sex of the individuals to be taken into account. Males carry only a single copy of the gene, meaning that allele frequencies in males translate directly into population allele frequency estimates. Precision in the terminology around female diagnostics is not always clear as not all methods consistently differentiated heterozygous from homozygous allele carriage. For reliability therefore, only data from males were included in these variant frequency maps. Data informing these maps therefore carried the additional inclusion criterion of disaggregating data according to sex.

**References**

1. Howes RE, Piel FB, Patil AP, Nyangiri OA, Gething PW, Hogg MM, Battle KE, Padilla CD, Baird JK, Hay SI: **G6PD deficiency prevalence and estimates of affected populations in malaria endemic countries: a geostatistical model-based map**. *PLoS Med* 2012, **9**:e1001339.

2. Mason PJ, Bautista JM, Gilsanz F: **G6PD deficiency: the genotype-phenotype association**. *Blood Rev* 2007, **21**:267-283.

3. Mourant AE, Kopec AC, Domaniewska-Sobczak K: **The Distribution of the Human Blood Groups and other Polymorphisms**. London: Oxford University Press; 1976.

4. Livingstone FB: **Frequencies of Hemoglobin Variants: Thalassemia, the Glucose-6-Phosphate Dehydrogenase Deficiency, G6PD Variants and Ovalocytosis in Human Populations**. New York: Oxford University Press; 1985.

5. Nkhoma ET, Poole C, Vannappagari V, Hall SA, Beutler E: **The global prevalence of glucose-6-phosphate dehydrogenase deficiency: a systematic review and meta-analysis**. *Blood Cells Mol Dis* 2009, **42**:267-278.

6. Singh S: **Distribution of certain polymorphic traits in populations of the Indian peninsula and South Asia**. *Isr J Med Sci* 1973, **9**:1225-1237.

7. Minucci A, Moradkhani K, Hwang MJ, Zuppi C, Giardina B, Capoluongo E: **Glucose-6-phosphate dehydrogenase (G6PD) mutations database: review of the "old" and update of the new mutations**. *Blood Cells Mol Dis* 2012, **48**:154-165.

8. Betke K, Brewer GJ, Kirkman HN, Luzzatto L, Motulsky AG, Ramot B, Siniscalco M: **Standardization of procedures for the study of glucose-6-phosphate dehydrogenase. Report of a WHO Scientific Group**. *World Health Organ Tech Rep Ser* 1967, **No. 366**:1-53.

9. Eziefula AC, Gosling R, Hwang J, Hsiang MS, Bousema T, Von Seidlein L, Drakeley C, on behalf of the Primaquine in Africa Discussion Group: **Rationale for short course primaquine in Africa to interrupt malaria transmission**. *Malar J* 2012, **11**:360.

10. Beutler E, Westwood B, Kuhl W: **Definition of the mutations of G6PD Wayne, G6PD Viangchan, G6PD Jammu, and G6PD 'LeJeune'**. *Acta Haematol* 1991, **86**:179-182.

11. Gething PW, Patil AP, Smith DL, Guerra CA, Elyazar IR, Johnston GL, Tatem AJ, Hay SI: **A new world malaria map: *Plasmodium falciparum* endemicity in 2010**. *Malar J* 2011, **10**:378.

12. Gething PW, Elyazar IR, Moyes CL, Smith DL, Battle KE, Guerra CA, Patil AP, Tatem AJ, Howes RE, Myers MF, George DB, Horby P, Wertheim HFL, Price RN, Mueller I, Baird JK, Hay SI: **A long neglected world malaria map: *Plasmodium vivax* endemicity in 2010**. *PLoS Negl Trop Dis* 2012, **6**:e1814.
