# Supplementary material for: Spatial distribution of G6PD deficiency variants across malaria-endemic regions
Source: Malar J. 2013 Nov 15;12:418. doi: 10.1186/1475-2875-12-418 (PMC3835423; doi:10.1186/1475-2875-12-418)
Supplement: Additional file 2 — Sources from which the datapoints in the maps were identified. [file 1475-2875-12-418-S2.docx]

**Additional Protocol 5 – Supplementary Bibliography**

Sources from which the datapoints included in the maps were identified included:

S1. Ademowo OG, Falusi AG (2002) Molecular epidemiology and activity of erythrocyte G6PD variants in a homogeneous Nigerian population. East Afr Med J 79: 42-44.

S2. Ainoon O, Boo NY, Yu YH, Cheong SK, Hamidah HN, et al. (2004) Complete molecular characterisation of glucose-6-phosphate dehydrogenase (G6PD) deficiency in a group of Malaysian Chinese neonates. Malays J Pathol 26: 89-98.

S3. Ainoon O, Joyce J, Boo NY, Cheong SK, Zainal ZA, et al. (1999) Glucose-6-phosphate dehydrogenase (G6PD) variants in Malaysian Chinese. Hum Mutat 14: 352.

S4. Ainoon O, Yu YH, Amir Muhriz AL, Boo NY, Cheong SK, et al. (2002) Glucose-6-phosphate dehydrogenase (G6PD) variants in Malaysian Malays. Hum Mutat 21: 101.

S5. Al-Allawi N, Eissa AA, Jubrael JM, Jamal SA, Hamamy H (2010) Prevalence and molecular characterization of Glucose-6-Phosphate dehydrogenase deficient variants among the Kurdish population of Northern Iraq. BMC Blood Disord 10: 6.

S6. Al-Jaouni SK, Jarullah J, Azhar E, Moradkhani K (2011) Molecular characterization of glucose-6-phosphate dehydrogenase deficiency in Jeddah, Kingdom of Saudi Arabia. BMC Res Notes 4: 436.

S7. Al-Musawi BM, Al-Allawi N, Abdul-Majeed BA, Eissa AA, Jubrael JM, et al. (2012) Molecular characterization of glucose-6-phosphate dehydrogenase deficient variants in Baghdad city - Iraq. BMC Blood Disord 12: 4.

S8. Alves J, Machado P, Silva J, Goncalves N, Ribeiro L, et al. (2010) Analysis of malaria associated genetic traits in Cabo Verde, a melting pot of European and sub Saharan settlers. Blood Cells Mol Dis 44: 62-68.

S9. Armoo S, Wilson M, Boakye D, Quakyi I (2010) Studies on ABO blood groups, haemoglobinopathies and G6PD genotypes, and *Plasmodium falciparum* infection in Kpone-on-Sea, Ghana. Am J Trop Med Hyg 83: 82-82.

S10. Badens C, Martinez di Montemuros F, Thuret I, Michel G, Mattei JF, et al. (2000) Molecular basis of haemoglobinopathies and G6PD deficiency in the Comorian population. Hematol J 1: 264-268.

S11. Beutler E, Kuhl W, Saenz GF, Rodriguez W (1991) Mutation analysis of glucose-6-phosphate dehydrogenase (G6PD) variants in Costa Rica. Hum Genet 87: 462-464.

S12. Bouanga JC, Mouele R, Prehu C, Wajcman H, Feingold J, et al. (1998) Glucose-6-phosphate dehydrogenase deficiency and homozygous sickle cell disease in Congo. Hum Hered 48: 192-197.

S13. Cai W, Filosa S, Martini G, Zhou Y, Zhou D, et al. (2001) [Molecular characterization of glucose-6-phosphate dehydrogenase deficiency in the Han and Li nationalities in Hainan, China and identification of a new mutation in human G6PD gene]. Zhonghua Yi Xue Yi Chuan Xue Za Zhi 18: 105-109.

S14. Cardoso MA, Scopel KK, Muniz PT, Villamor E, Ferreira MU (2012) Underlying factors associated with anemia in Amazonian children: a population-based, cross-sectional study. PLoS One 7: e36341.

S15. Chalvam R, Colah RB, Mohanty D, Ghosh K, Mukherjee MB (2009) Molecular heterogeneity of glucose-6-phosphate dehydrogenase deficiency among the tribals in Western India. Blood Cells Mol Dis 43: 156-157.

S16. Chalvam R, Kedar PS, Colah RB, Ghosh K, Mukherjee MB (2008) A novel R198H mutation in the glucose-6-phosphate dehydrogenase gene in the tribal groups of the Nilgiris in Southern India. J Hum Genet 53: 181-184.

S17. Chalvam R, Mukherjee MB, Colah RB, Mohanty D, Ghosh K (2007) G6PD Namoru (208 T--> C) is the major polymorphic variant in the tribal populations in southern India. Br J Haematol 136: 512-513.

S18. Chen HL, Huang MJ, Huang CS, Tang TK (1997) Two novel glucose 6-phosphate dehydrogenase deficiency mutations and association of such mutations with F8C/G6PD haplotype in Chinese. J Formos Med Assoc 96: 948-954.

S19. Chiang SH, Wu SJ, Wu KF, Hsiao KJ (1999) Neonatal screening for glucose-6-phosphate dehydrogenase deficiency in Taiwan. Southeast Asian J Trop Med Public Health 30 Suppl 2: 72-74.

S20. Chiu DTY, Zuo L, Chao L, Chen E, Louie E, et al. (1993) Molecular characterization of glucose-6-phosphate dehydrogenase (G6PD) deficiency in patients of Chinese descent and identification of new base substitutions in the human G6PD gene. Blood 81: 2150-2154.

S21. Clark TG, Fry AE, Auburn S, Campino S, Diakite M, et al. (2009) Allelic heterogeneity of G6PD deficiency in West Africa and severe malaria susceptibility. Eur J Hum Genet 17: 1080-1085.

S22. Cossio-Gurrola G, Arambula-Meraz E, Perea M, Garcia N, Correa AS, et al. (2010) Glucose-6-phosphate dehydrogenase (G6PD) molecular variant deficiency: identification in Panama pediatric population. Blood Cells Mol Dis 44: 115-116.

S23. Coulibaly FH, Koffi G, Toure HA, Bouanga JC, Allangba O, et al. (2000) Molecular genetics of glucose-6-phosphate dehydrogenase deficiency in a population of newborns from Ivory Coast. Clin Biochem 33: 411-413.

S24. Crompton PD, Traore B, Kayentao K, Doumbo S, Ongoiba A, et al. (2008) Sickle cell trait is associated with a delayed onset of malaria: Implications for time-to-event analysis in clinical studies of malaria. JID 198: 1265-1275.

S25. De Araujo C, Migot-Nabias F, Guitard J, Pelleau S, Vulliamy T, et al. (2006) The role of the G6PD A-376G/968C allele in glucose-6-phosphate dehydrogenase deficiency in the seerer population of Senegal. Haematologica 91: 262-263.

S26. Du C, He Y (1997) [A case of nt 1004C --> A G6PD gene mutation in Yunnan Han people]. Zhonghua Xue Ye Xue Za Zhi 18: 535-537.

S27. Francine MR (2000) Molecular analysis for the detection of glucose-6-phosphate dehydrogenase (G6PD) deficiency. Yogyakarta: Gadjah Mada University (Thesis).

S28. Gari MA, Chaudhary AG, Al-Qahtani MH, Abuzenadah AM, Waseem A, et al. (2010) Frequency of Mediterranean mutation among a group of Saudi G6PD patients in Western region-Jeddah. Int J Lab Hematol 32: 17-21.

S29. Guindo A, Fairhurst RM, Doumbo OK, Wellems TE, Diallo DA (2007) X-linked G6PD deficiency protects hemizygous males but not heterozygous females against severe malaria. PLoS Medicine 4: 516-522.

S30. Hamel AR, Cabral IR, Sales TS, Costa FF, Olalla Saad ST (2002) Molecular heterogeneity of G6PD deficiency in an Amazonian population and description of four new variants. Blood Cells Mol Dis 28: 399-406.

S31. Hirono A, Ishii A, Kere N, Fujii H, Hirono K, et al. (1995) Molecular analysis of glucose-6-phosphate dehydrogenase variants in the Solomon Islands. Am J Hum Genet 56: 1243-1245.

S32. Huang CS, Hung KL, Huang MJ, Li YC, Liu TH, et al. (1996) Neonatal jaundice and molecular mutations in glucose-6-phosphate dehydrogenase deficient newborn infants. Am J Hematol 51: 19-25.

S33. Hung NM, Eto H, Mita T, Tsukahara T, Hombhanje FW, et al. (2008) Glucose - 6 - Phosphate Dehydrogenase (G6PD) variants in East Sepik Province of Papua New Guinea : G6PD Jammu, G6PD Vanua Lava, and a novel variant (G6PD Dagua). Trop Med Health 36: 163-169.

S34. Iwai K, Hirono A, Matsuoka H, Kawamoto F, Horie T, et al. (2001) Distribution of glucose-6-phosphate dehydrogenase mutations in Southeast Asia. Hum Genet 108: 445-449.

S35. Jalloh A, Jalloh M, Gamanga I, Baion D, Sahr F, et al. (2008) G6PD deficiency assessment in Freetown, Sierra Leone, reveals further insight into the molecular heterogeneity of G6PD A. J Hum Genet 53: 675-679.

S36. Jiang W, Yu G, Liu P, Geng Q, Chen L, et al. (2006) Structure and function of glucose-6-phosphate dehydrogenase-deficient variants in Chinese population. Hum Genet 119: 463-478.

S37. Jiang WY, Zhou BY, Yu GL, Liu H, Zeng JB, et al. (2012) G6PD genotype and its associated enzymatic activity in a Chinese population. Biochem Genet 50: 34-44.

S38. Johnson MK, Clark TD, Njama-Meya D, Rosenthal PJ, Parikh S (2009) Impact of the method of G6PD deficiency assessment on genetic association studies of malaria susceptibility. PLoS One 4: e7246.

S39. Kaeda JS, Chhotray GP, Ranjit MR, Bautista JM, Reddy PH, et al. (1995) A new glucose-6-phosphate dehydrogenase variant, G6PD Orissa (44 Ala-->Gly), is the major polymorphic variant in tribal populations in India. Am J Hum Genet 57: 1335-1341.

S40. Karimi M, Yavarian M, Afrasiabi A, Dehbozorgian J, Rachmilewitz E (2008) Prevalence of beta-thalassemia trait and glucose-6-phosphate dehydrogenase deficiency in Iranian Jews. Arch Med Res 39: 212-214.

S41. Kempinska-Podhorodecka A, Knap O, Drozd A, Kaczmarczyk M, Parafiniuk M, et al. (2013) Analysis of the genetic variants of glucose-6-phosphate dehydrogenase in inhabitants of the 4th Nile cataract region in Sudan. Blood Cells Mol Dis 50: 115-118.

S42. Keskin N, Ozdes I, Keskin A, Acikbas I, Bagci H (2002) Incidence and molecular analysis of glucose-6-phosphate dehydrogenase deficiency in the province of Denizli, Turkey. Med Sci Monit 8: CR453-456.

S43. Kim S, Nguon C, Guillard B, Duong S, Chy S, et al. (2011) Performance of the CareStart G6PD deficiency screening test, a point-of-care diagnostic for primaquine therapy screening. PLoS One 6: e28357.

S44. Leslie T, Moiz B, Mohammad N, Amanzai O, Rashid H, et al. (2013) Prevalence and molecular basis of glucose-6-phosphate dehydrogenase deficiency in Afghan populations: implications for treatment policy in the region. Malar J 12: 230.

S45. Louicharoen C, Nuchprayoon I (2005) G6PD Viangchan (871G>A) is the most common G6PD-deficient variant in the Cambodian population. J Hum Genet 50: 448-452.

S46. Matsuoka H, Arai M, Yoshida S, Tantular IS, Pusarawati S, et al. (2003) Five different glucose-6-phophate [correction phosphate]dehydrogenase (G6PD) variants found among 11 G6PD-deficient persons in Flores Island, Indonesia. J Hum Genet 48: 541-544.

S47. Matsuoka H, Jichun W, Hirai M, Yoshida S, Arai M, et al. (2003) Two cases of glucose-6-phosphate dehydrogenase-deficient Nepalese belonging to the G6PD Mediterranean-type, not India-Pakistan sub-type but Mediterranean-Middle East sub-type. J Hum Genet 48: 275-277.

S48. Matsuoka H, Wang J, Hirai M, Arai M, Yoshida S, et al. (2004) Glucose-6-phosphate dehydrogenase (G6PD) mutations in Myanmar: G6PD Mahidol (487G>A) is the most common variant in the Myanmar population. J Hum Genet 49: 544-547.

S49. May J, Meyer CG (2003) A synonymous mutation of ancient origin in the glucose-6-phosphate dehydrogenase gene and assessment of haplotypes. Blood Cells Mol Dis 30: 144-145.

S50. Meissner PE, Coulibaly B, Mandi G, Mansmann U, Witte S, et al. (2005) Diagnosis of red cell G6PD deficiency in rural Burkina Faso: comparison of a rapid fluorescent enzyme test on filter paper with polymerase chain reaction based genotyping. Br J Haematol 131: 395-399.

S51. Millimono TS, Loua KM, Rath SL, Relvas L, Bento C, et al. (2012) High prevalence of hemoglobin disorders and glucose-6-phosphate dehydrogenase (G6PD) deficiency in the Republic of Guinea (West Africa). Hemoglobin 36: 25-37.

S52. Miri-Moghaddam E, Mortazavi Y, Nakhaee A, Khazaei Feizabad A (2013) Prevalence and molecular identification of the mediterranean variant among G6PD-deficient Sistani and Balouch males in Southeastern Iran. Biochem Genet 51: 131-138.

S53. Mombo LE, Ntoumi F, Bisseye C, Ossari S, Lu CY, et al. (2003) Human genetic polymorphisms and asymptomatic *Plasmodium falciparum* malaria in Gabonese schoolchildren. Am J Trop Med Hyg 68: 186-190.

S54. Mortazavi Y, Mirzamohammadi F, Ardestani MT, Mirimoghadam E, Vulliamy TJ (2010) Glucose 6-phosphate dehydrogenase deficiency in Tehran, Zanjan and sistan-balouchestan provinces: Prevalence and frequency of Mediterranean variant of G6PD. Iran J Biotechnol 8: 229-233.

S55. Nadarajan V, Shanmugam H, Sthaneshwar P, Jayaranee S, Sultan KS, et al. (2011) Modification to reporting of qualitative fluorescent spot test results improves detection of glucose-6-phosphate dehydrogenase (G6PD)-deficient heterozygote female newborns. Int J Lab Hematol 33: 463-470.

S56. Nakhaee A, Salimi S, Zadehvakili A, Dabiri S, Noora M, et al. (2012) The Prevalence of Mediterranean Mutation of Glucose-6-Phosphate Dehydrogenase (G6PD) in Zahedan. Zahedan Journal of Research in Medical Sciences 14: 39-43.

S57. Neto JPD, Dourado MV, dos Reis MG, Goncalves MS (2008) A novel c. 197T -> A variant among Brazilian neonates with glucose-6-phosphate dehydrogenase deficiency. Genet Mol Biol 31: 33-35.

S58. Nezhad SRK, Mashayekhi A, Khatami SR, Daneshmand S, Fahmi F, et al. (2009) Prevalence and molecular identification of Mediterranean glucose-6-phosphate dehydrogenase deficiency in Khuzestan Province, Iran. Iran J Public Health 38: 127-131.

S59. Ninokata A, Kimura R, Samakkarn U, Settheetham-Ishida W, Ishida T (2006) Coexistence of five G6PD variants indicates ethnic complexity of Phuket islanders, Southern Thailand. J Hum Genet 51: 424-428.

S60. Nishank SS, Chhotray GP, Kar SK, Ranjit MR (2008) Molecular variants of G6PD deficiency among certain tribal communities of Orissa, India. Ann Hum Biol 35: 355-361.

S61. Nuchprayoon I, Louicharoen C, Charoenvej W (2008) Glucose-6-phosphate dehydrogenase mutations in Mon and Burmese of southern Myanmar. J Hum Genet 53: 48-54.

S62. Nuchprayoon I, Sanpavat S, Nuchprayoon S (2002) Glucose-6-phosphate dehydrogenase (G6PD) mutations in Thailand: G6PD Viangchan (871G>A) is the most common deficiency variant in the Thai population. Hum Mutat 19: 185.

S63. Oliveira RAG, Oshiro M, Hirata MH, Hirata RDC, Ribeiro GS, et al. (2009) A novel point mutation in a class IV glucose-6-phosphate dehydrogenase variant (G6PD Sao Paulo) and polymorphic G6PD variants in Sao Paulo State, Brazil. Genet Mol Biol 32: 251-254.

S64. Othman A, Wong F, Boo N, Wang M, Nh H (2008) Rapid molecular screening of G6PD variants in Malaysian Chinese newborns using Taqman MGB SNP assay. Int J Lab Hematol. pp. (Suppl 1) 125-125.

S65. Pan M, Lin M, Yang L, Wu J, Zhan X, et al. (2013) Glucose-6-phosphate dehydrogenase (G6PD) gene mutations detection by improved high-resolution DNA melting assay. Mol Biol Rep 40: 3073-3082.

S66. Phompradit P, Kuesap J, Chaijaroenkul W, Rueangweerayut R, Hongkaew Y, et al. (2011) Prevalence and distribution of glucose-6-phosphate dehydrogenase (G6PD) variants in Thai and Burmese populations in malaria endemic areas of Thailand. Malar J 10: 368.

S67. Qi XL, Shan K, Xie Y, Wu CX, Xiu J, et al. (2006) Study on the mutations of G6PD gene in Dong ethnic group in Guizhou Congjiang. Chin J Endemiology 25: 283-285.

S68. Rahimi Z, Vaisi-Raygani A, Nagel RL, Muniz A (2006) Molecular characterization of glucose-6-phosphate dehydrogenase deficiency in the Kurdish population of Western Iran. Blood Cells Mol Dis 37: 91-94.

S69. Ren X, He Y, Du C, Jiang W, Chen L, et al. (2001) A novel mis-sense mutation (G1381A) in the G6PD gene identified in a Chinese man. Chin Med J (Engl) 114: 399-401.

S70. Saad ST, Salles TS, Carvalho MH, Costa FF (1997) Molecular characterization of glucose-6-phosphate dehydrogenase deficiency in Brazil. Hum Hered 47: 17-21.

S71. Saha N, Ramzan M, Tay JS, Low PS, Basair JB, et al. (1994) Molecular characterisation of red cell glucose-6-phosphate dehydrogenase deficiency in north-west Pakistan. Hum Hered 44: 85-89.

S72. Sakuntabhai A (2013) Unpublished data from Madagascar.

S73. Santana MS, Monteiro WM, Siqueira AM, Costa MF, Sampaio V, et al. (2013) Glucose-6-phosphate dehydrogenase deficient variants are associated with reduced susceptibility to malaria in the Brazilian Amazon. Trans R Soc Trop Med Hyg.

S74. Sarkar S, Biswas NK, Dey B, Mukhopadhyay D, Majumder PP (2010) A large, systematic molecular-genetic study of G6PD in Indian populations identifies a new non-synonymous variant and supports recent positive selection. Infect Genet Evol 10: 1228-1236.

S75. Satyagraha AW (2013) Unpublished data from Indonesia.

S76. Shah SS, Macharia A, Makale J, Uyoga S, Kivinen K, et al. (2013) Genetic determinants of glucose-6-phosphate dehydrogenase activity in Kenya. Unpublished work.

S77. Soemantri AG, Saha S, Saha N, Tay JS (1995) Molecular variants of red cell glucose-6-phosphate dehydrogenase deficiency in Central Java, Indonesia. Hum Hered 45: 346-350.

S78. Soewono S, Martini T, Shirakawa T, Nishiyama K (2000) Glucose-6-phosphate dehydrogenase (G6PD) deficiency variants in small isolated islands in eastern Indonesia. Jurnal Kedokteran Yarsi 8: 87-92.

S79. Suchdev PS, Ruth LJ, Earley M, Macharia A, Williams TN (2012) The burden and consequences of inherited blood disorders among young children in western Kenya. Matern Child Nutr.

S80. Sukumar S, Mukherjee MB, Colah RB, Mohanty D (2004) Molecular basis of G6PD deficiency in India. Blood Cells Mol Dis 33: 141-145.

S81. Tang TK, Huang WY, Tang CJ, Hsu M, Cheng TA, et al. (1995) Molecular basis of glucose-6-phosphate dehydrogenase (G6PD) deficiency in three Taiwan aboriginal tribes. Hum Genet 95: 630-632.

S82. Tantular IS, Matsuoka H, Kasahara Y, Pusarawati S, Kanbe T, et al. (2010) Incidence and mutation analysis of glucose-6-phosphate dehydrogenase deficiency in eastern Indonesian populations. Acta Med Okayama 64: 367-373.

S83. Than AM, Harano T, Harano K, Myint AA, Ogino T, et al. (2005) High incidence of 3-thalassemia, hemoglobin E, and glucose-6-phosphate dehydrogenase deficiency in populations of malaria-endemic southern Shan State, Myanmar. Int J Hematol 82: 119-123.

S84. Tseng CP, Huang CL, Chong KY, Hung IJ, Chiu DT (2005) Rapid detection of glucose-6-phosphate dehydrogenase gene mutations by denaturing high-performance liquid chromatography. Clin Biochem 38: 973-980.

S85. Vaca G, Arambula E, Esparza A (2002) Molecular heterogeneity of glucose-6-phosphate dehydrogenase deficiency in Mexico: overall results of a 7-year project. Blood Cells Mol Dis 28: 436-444.

S86. Wang J, Matsuoka H, Hirai M, Mu L, Yang L, et al. (2010) The first case of a class I glucose-6-phosphate dehydrogenase deficiency, G6PD Santiago de Cuba (1339 GA), in a Chinese population as found in a survey for G6PD deficiency in Northeastern and Central China. Acta Med Okayama 64: 49-54.

S87. Wu CX, He Y, Shan KR, Li Y, Xiu J, et al. (2006) Study the mutations of glucose-6-phosphate dehydrogenase gene in Yao ethnic group in Guizhou Libo. Chin J Endemiology 25: 402-404.

S88. Wu CX, Shan KR, He Y, Qi XL, Li Y, et al. (2007) Detection of glucose-6-phosphate dehydrogenase gene mutations of Tujia ehtnic in Jiangkou, Guizhou. Chin J Endemiology 26: 415-417.

S89. Xiu J, Qi XL, Shan KR, Xie Y, He Y, et al. (2005) [G6PD Gene Mutations in Shui people in Sandu of Guizhou]. Zhongguo Shi Yan Xue Ye Xue Za Zhi 13: 147-150.

S90. Xu W, Westwood B, Bartsocas CS, Malcorra-Azpiazu JJ, Indrak K, et al. (1995) Glucose-6 phosphate dehydrogenase mutations and haplotypes in various ethnic groups. Blood 85: 257-263.

S91. Yan JB, Xu HP, Xiong C, Ren ZR, Tian GL, et al. (2010) Rapid and reliable detection of glucose-6-phosphate dehydrogenase (G6PD) gene mutations in Han Chinese using high-resolution melting analysis. J Mol Diagn 12: 305-311.

S92. Yan T, Cai R, Mo O, Zhu D, Ouyang H, et al. (2006) Incidence and complete molecular characterization of glucose-6-phosphate dehydrogenase deficiency in the Guangxi Zhuang autonomous region of southern China: description of four novel mutations. Haematologica 91: 1321-1328.

S93. Yang Y, Zhu Y, Li D, Li Z, Lu H, et al. (2007) Characterization of glucose-6-phosphate dehydrogenase deficiency and identification of a novel haplotype 487G>A/IVS5-612(G>C) in the Achang population of Southwestern China. Sci China C Life Sci 50: 479-485.
