# Supplementary figures and images for: Spatial distribution of G6PD deficiency variants across malaria-endemic regions
Source: Malar J. 2013 Nov 15;12:418. doi: 10.1186/1475-2875-12-418 (PMC3835423; doi:10.1186/1475-2875-12-418)

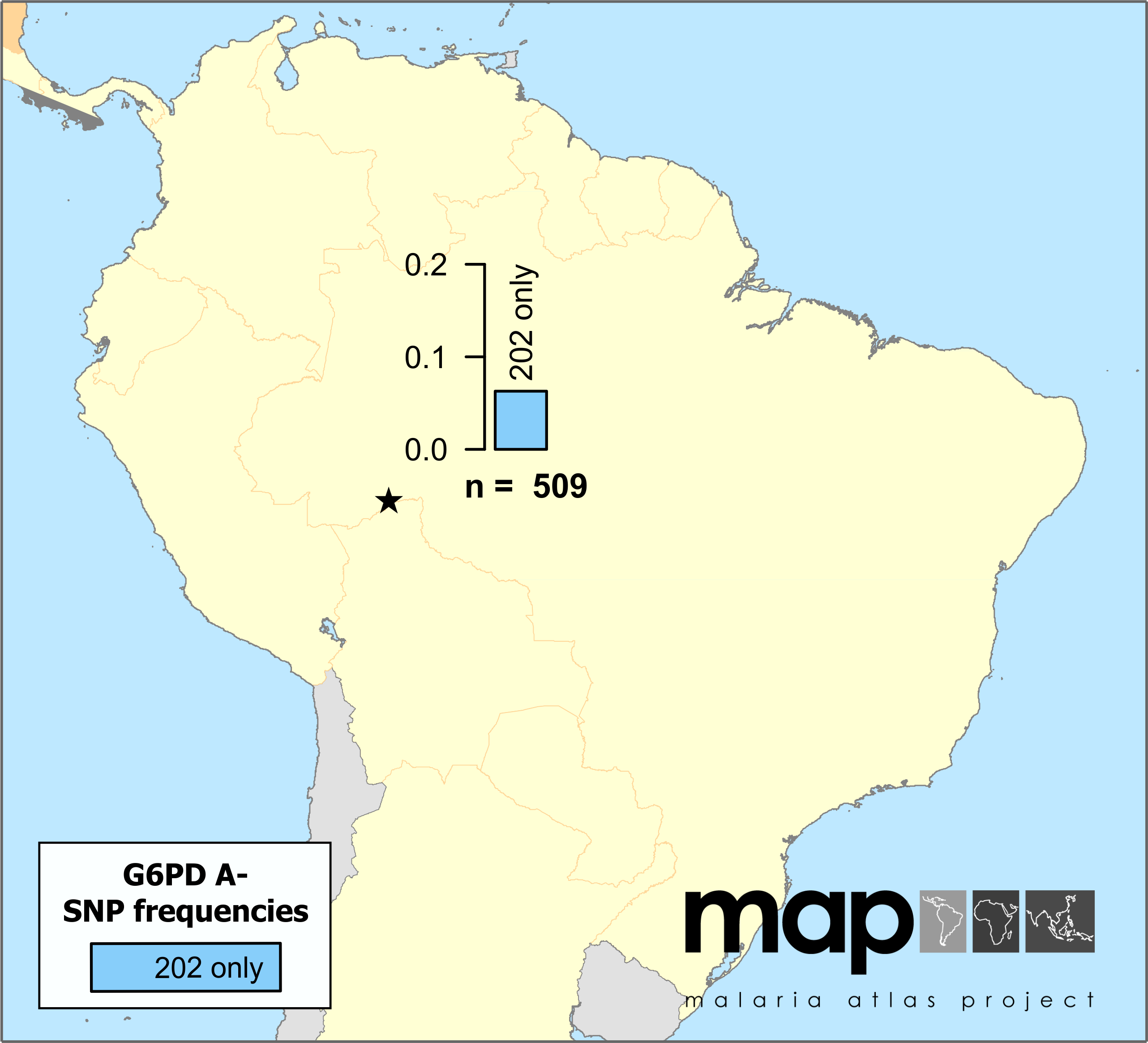

Supplement: Additional file 3 — Map series 2: Americas. Bar charts represent population surveys which examined the frequencies of selected G6PD variants in representative population samples. These population groups had not undergone prior G6PDd phenotype screening. The variants which were tested for in each location are listed above the x-axis. Survey locations are indicated by nearby black stars. Sample size is stated under each plot. [file 1475-2875-12-418-S3.tif]

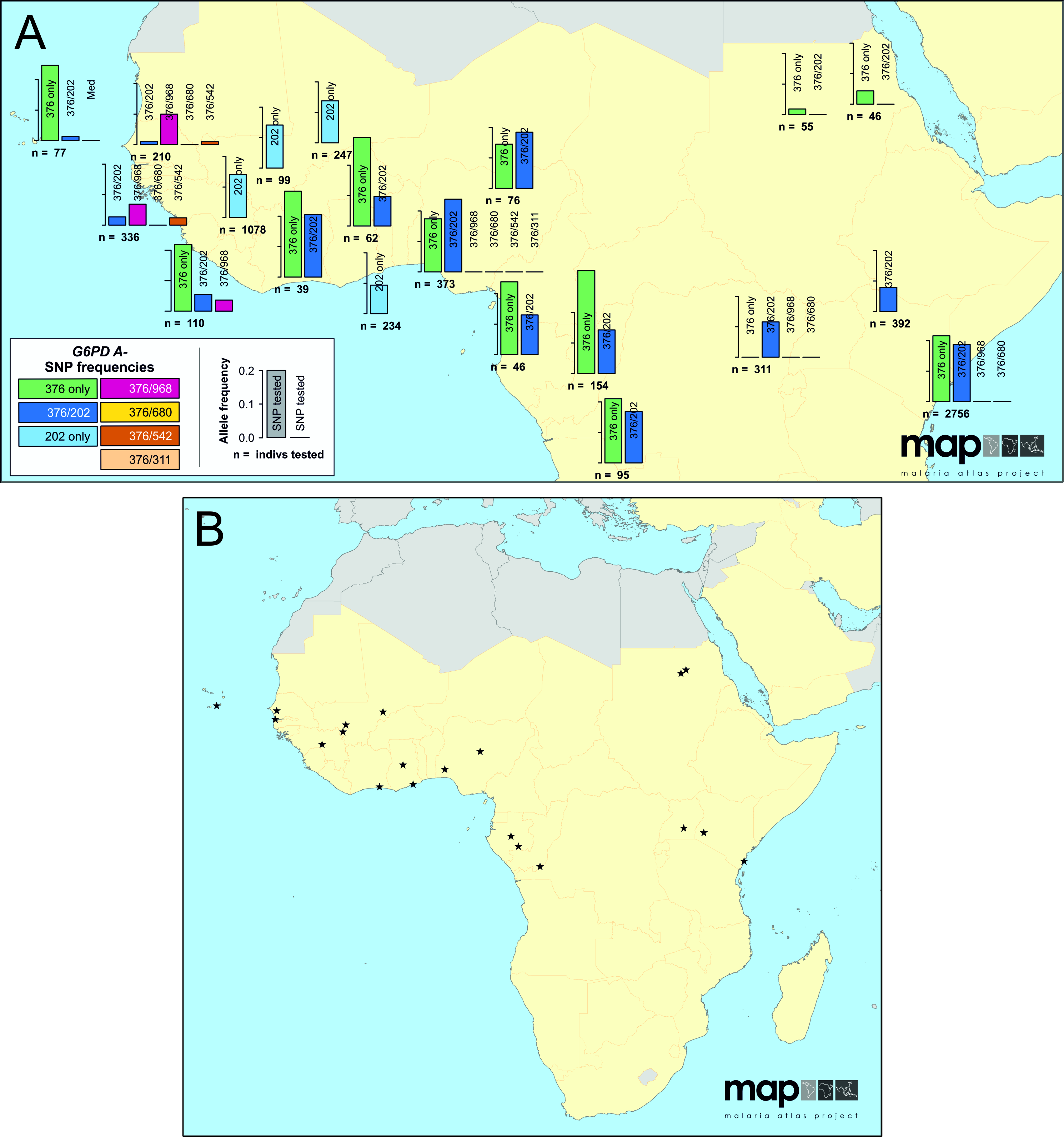

Supplement: Additional file 4 — Map series 2: Africa. (A) Bar charts represent population surveys which examined the frequencies of selected G6PD variants in representative population samples. These population groups had not undergone prior G6PDd phenotype screening. The variants which were tested for in each location are listed above the x-axis. Sample size is stated under each plot. Survey locations are mapped to closest approximation at the point of origin of the plots; exact survey locations are shown in Panel B. (Panel A is reproduced from Figure 8 in the main manuscript). [file 1475-2875-12-418-S4.tif]

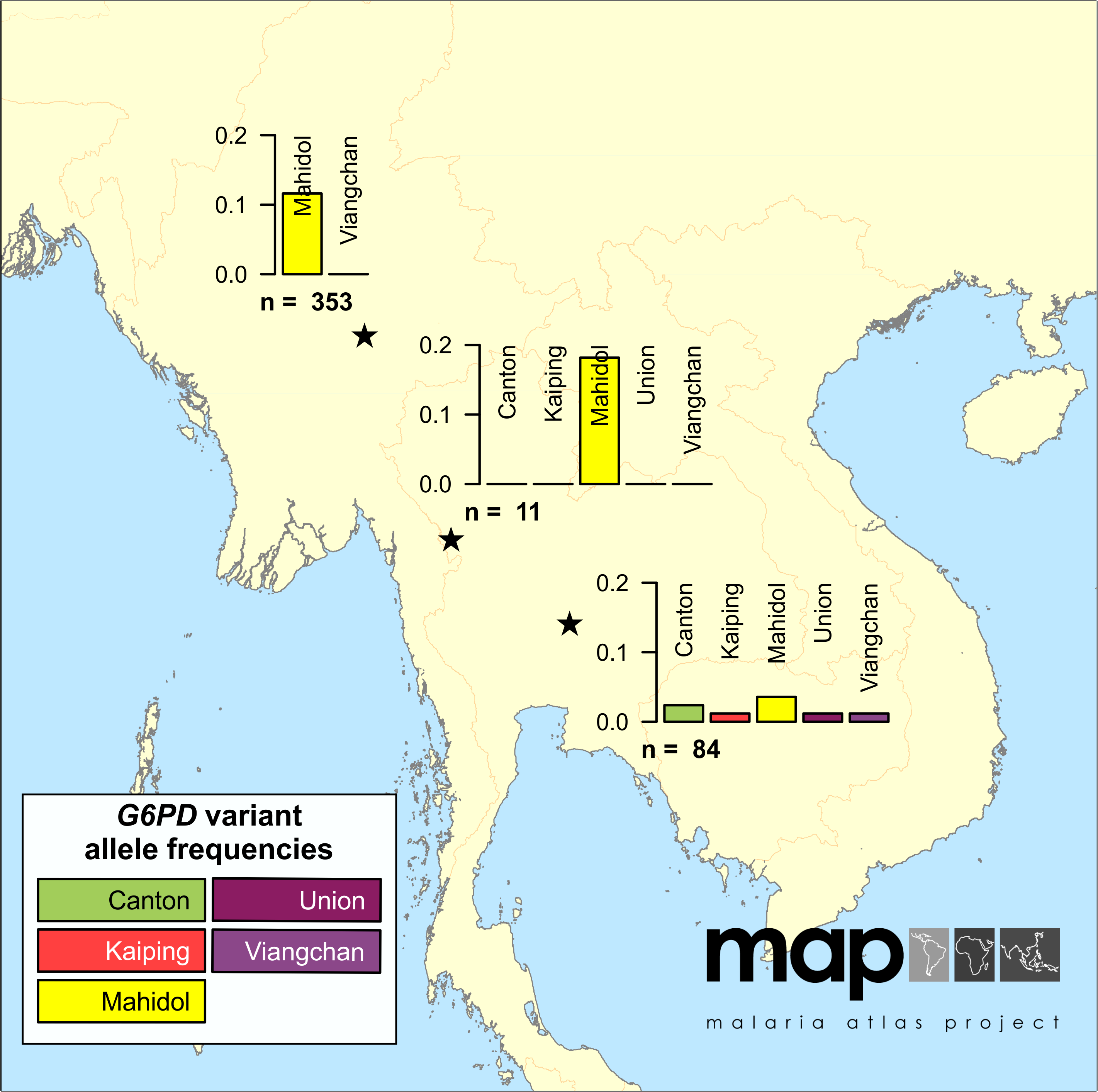

Supplement: Additional file 5 — Map series 2: Asia. Bar charts represent population surveys which examined the frequencies of selected G6PD variants in representative population samples. These population groups had not undergone prior G6PDd phenotype screening. The variants which were tested for in each location are listed above the x-axis. Survey locations are indicated by nearby black stars. Sample size is stated under each plot. [file 1475-2875-12-418-S5.tif]
